# Ptprz1b phosphatase binds Prickle2 to promote its membrane localization

**DOI:** 10.1016/j.isci.2026.116707

**Published:** 2026-07-13

**Authors:** Yao Le, Sarka Novotna, Lorena Agostini Maia, Nicholas S. Tolwinski, Christoph Winkler, Jakub Harnos

**Affiliations:** 1 Department of Biological Sciences and Centre for Bioimaging Sciences, National University of Singapore, Singapore 117543, Singapore; 2 Department of Experimental Biology, Faculty of Science, Masaryk University, 62500 Brno, Czechia; 3 Programme in Cancer and Stem Cell Biology, Duke-NUS Medical School, Singapore 169857, Singapore

**Keywords:** Planar cell polarity signaling, Vangl/Prickle, protein tyrosine phosphatase receptor, membrane localization

## Abstract

The Wnt/planar cell polarity (PCP) pathway plays a critical role in the development and homeostasis of multicellular organisms. Molecularly, it is organized into two core protein complexes, Vangl/Prickle and Dishevelled/Frizzled. Here, we identify the receptor-type tyrosine phosphatase Ptprz1b as a regulator of Prickle membrane retention. Ptprz1b binds Prickle2 through multiple regions and depends on Vangl through formation of a membrane-competent Prickle2 pool rather than direct Ptprz1b-Vangl binding. Loss of *ptprz1b* impairs Prickle2 membrane localization in zebrafish embryos and increases its turnover at the plasma membrane, while membrane Vangl2 levels remain unchanged. A catalytic trapping mutant of Ptprz1b shows increased Prickle2 binding but reduced membrane retention, supporting an activity-dependent stabilization mechanism. Ptprz1b deficiency leads to defects in PCP-dependent morphogenetic processes, including impaired convergent extension in zebrafish and neural tube closure defects in *Xenopus*. Together, these findings identify Ptprz1b as a regulator of membrane-associated Prickle2 required for PCP-dependent morphogenesis *in vivo*.

## Highlights

• Ptprz1b binds Prickle proteins through multiple interaction regions
• Vangl proteins enable Ptprz1b-Prickle complex formation
• Ptprz1b stabilizes Prickle at the plasma membrane *in vivo*
• Ptprz1b regulates vertebrate PCP-dependent morphogenesis

## Graphical abstract

**Figure:**
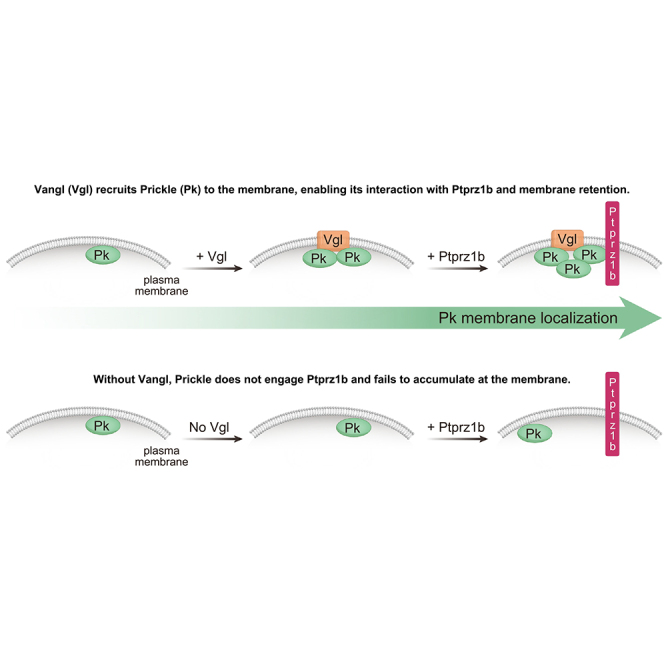

## Teaser

Molecular biology; Cell biology

## Introduction

Wnt signaling plays a critical role in regulating developmental processes, tissue homeostasis, and disease mechanisms, including cancer and congenital defects.^1^^,^^2^^,^^3^^,^^4^ Among the diverse Wnt pathways, the non-canonical planar cell polarity (PCP) pathway stands out as a central mechanism for coordinating planar organization of cells within tissues, ensuring directional behavior, structural integrity, and the emergence of complex form during vertebrate development.^5^^,^^6^^,^^7^^,^^8^ The PCP pathway relies on the asymmetric distribution of Frizzled/Dishevelled (Fz/Dvl) and Vangl/Prickle protein complexes at the plasma membrane, which serves as a critical platform for assembling these complexes and generating polarized signaling output.^9^^,^^10^ The activation of Wnt pathways is tightly controlled by extracellular modulators, including agonists like R-spondins, Norrin, and GPCRs (e.g., Gpr124), and antagonists such as Dkks, Sfrps, and the E3 ubiquitin ligase RNF43, which inhibit Wnt activity by targeting pathway components.^11^^,^^12^^,^^13^^,^^14^

While the Fz/Dvl complex has been extensively characterized in both canonical and non-canonical signaling,^15^^,^^16^^,^^17^^,^^18^^,^^19^^,^^20^^,^^21^ the Vangl/Prickle complex remains comparatively less understood, despite its essential role in tissue polarity and polarized cell behaviors.^22^^,^^23^ Recent work has begun to shed light on mechanisms regulating VANGL2 protein stability, including degradation by the KBTBD7-VCP-Cul3 complex, a process antagonized by Wnt5A-mediated phosphorylation,^24^ and stabilization via CK1δ/ε-dependent phosphorylation.^25^^,^^26^ Other proteins have been suggested to influence VANGL2 behavior. For instance, Ryk, an atypical Wnt receptor, was shown to interact with VANGL2, leading to the increase of its stability.^27^^,^^28^ Similarly, Rack1 was implicated in VANGL2 membrane localization,^29^ but whether it directly stabilizes the Vangl/Prickle complex or acts more broadly as a scaffolding factor remains unresolved. Importantly, these studies do not address the co-stabilization of the Vangl/Prickle complex as a functional unit, nor do they identify membrane-associated factors that actively promote its persistence at the cell cortex.

PTPRZ1 (PTPRZ1 in humans, Ptprz1 in *Xenopus*, and Ptprz1b in zebrafish) is a receptor-like protein tyrosine phosphatase implicated in human cerebral cortex development and glioma metastasis.^30^^,^^31^^,^^32^^,^^33^^,^^34^^,^^35^ It facilitates glioma metastasis by activating Rho/ROCK, a well-known effector downstream of Wnt/PCP that induces polarized cytoskeleton remodeling.^35^ In addition, we have recently shown that deficiencies in zebrafish neurulation induced by a Ptprz1b mutation can be rescued by Prickle overexpression.^36^ However, how Ptprz1 signaling interacts with the Wnt/PCP pathway remains unexplored.

In this study, using proteomic approaches and high-resolution live imaging, we identified Ptprz1b as a key membrane-associated regulator of Wnt/PCP signaling that is required for Prickle2 retention at the plasma membrane in zebrafish embryos. We further show that Ptprz1b plays an essential role in convergent extension movements in zebrafish and neural plate folding in *Xenopus*, which are both classical PCP readouts. In *Drosophila melanogaster*, knockdown of the Ptprz1b homolog PTP99A did not affect PCP in the wing, suggesting that this regulatory mechanism is likely vertebrate-specific. Importantly, these functions depend on Vangl, supporting a model in which Ptprz1b cooperates with core PCP components to control Prickle membrane localization and polarized cell behavior during vertebrate gastrulation and neurulation.

## Results

### Prickle2 interacts via its N-terminal, central, and C-terminal regions with Ptprz1b

**Figure 1:**
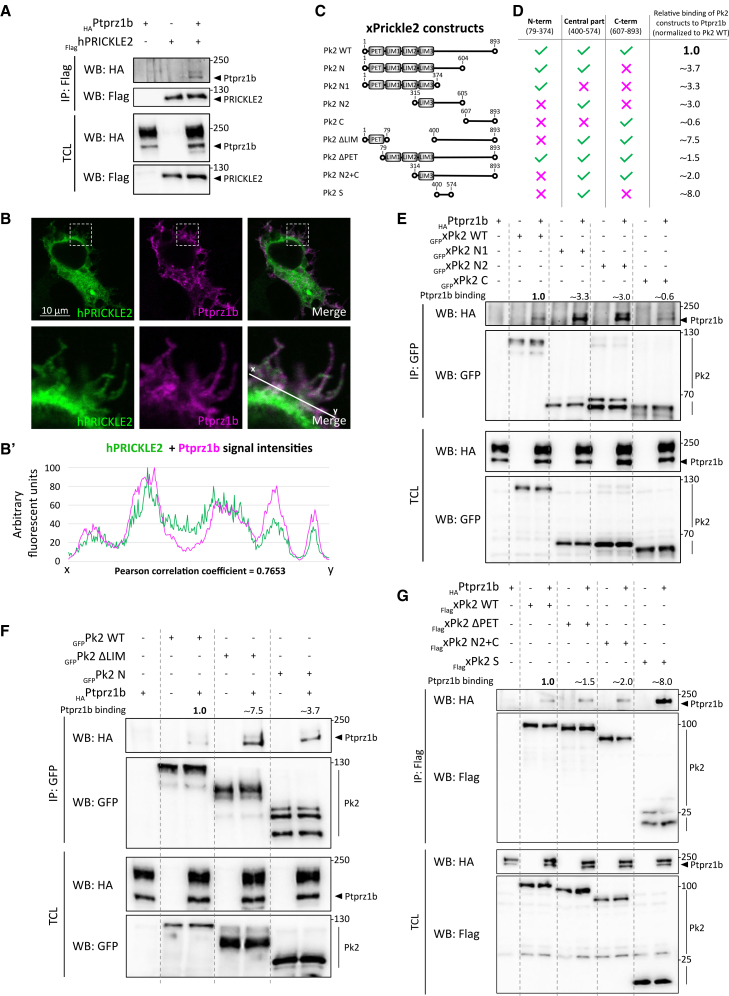
Prickle2 interacts via its N-terminal, central, and C-terminal regions with Ptprz1b (A) Co-immunoprecipitation (IP) of HA-tagged Ptprz1b with FLAG-tagged human PRICKLE2 in HEK293 cells. Input and IP samples were analyzed by immunoblotting. Ptprz1b strongly associates with PRICKLE2. (B) Representative immunofluorescence images, the zoomed area highlighted by the white box below, and their signal quantification (the white line *x*-*y*) in B′ showing that co-expression of Ptprz1b with EGFP-hPRICKLE2 leads to their enrichment in membrane protrusions in HEK293 cells. (C) Schematic representation of *Xenopus* Prickle2 truncation constructs used in this study. (D) Domain organization of Prickle2 highlighting the N-terminal (LIM-containing), central, and C-terminal regions, and their relative binding capacities normalized to full-length Prickle2 (WT) based on co-IP analyses in (E)–(G). (E–G) Co-IP assays of Ptprz1b with Prickle2 truncation mutants. All major regions of Prickle2 retain the ability to interact with Ptprz1b, indicating multiple binding interfaces. Deletion constructs frequently exhibit stronger binding than full-length Prickle2, suggesting that the interaction is conformationally regulated.

To test whether Ptprz1b can physically associate with Prickle family proteins, we performed immunoprecipitation assays in HEK293 cells. These cells lack endogenous PTPRZ1 expression, according to the Human Protein Atlas (https://www.proteinatlas.org/), and we could, therefore, employ a previously validated overexpression construct of zebrafish Ptprz1b^36^ for our interaction assays. We observed that overexpressed zebrafish Ptprz1b co-immunoprecipitated with human PRICKLE1 and PRICKLE2 (Figure 1A; Figures S1A and S1B). This suggested an interaction pattern among both PRICKLE paralogs.

We next analyzed the subcellular localization of these proteins. When co-expressed with Ptprz1b, both PRICKLE1 and PRICKLE2 were efficiently recruited into plasma membrane protrusions (Figure 1B-B’; Figure S1D), contrasting with their diffusive cytoplasmic distribution when expressed alone (Figure S1C).

To map the regions of Prickle2 responsible for interaction with Ptprz1b, we generated a panel of deletion variants of *Xenopus* Prickle2^37^ and tested them by co-immunoprecipitation (Figures 1C–1G). All major regions of Prickle2 could bind Ptprz1b, indicating that the interaction is not restricted to a single region. Specifically, we identified three interaction regions: the N-terminal region (aa 79–374, containing the LIM1–3 domains), the central region (aa 400–574), and the C-terminal region (aa 607–893) (Figures 1C–1G). While all deletion constructs showed stronger interaction in biochemical assays than Prickle2 WT, the isolated C-terminal fragment itself displayed weak interaction (Figure 1E). This is likely due to its predominant nuclear localization, which may limit its availability for membrane-associated Ptprz1b binding (Figure S2C).

Together, these data indicate that Prickle2 interacts with Ptprz1b through three distinct regions. The reduced binding of the full-length protein compared with deletion mutants suggests that the full-length Prickle2 protein adopts a conformation in which binding sites to Ptprz1b are partially masked.

### Prickle2 N terminus interacts with its C terminus to regulate its subcellular localization

**Figure 2:**
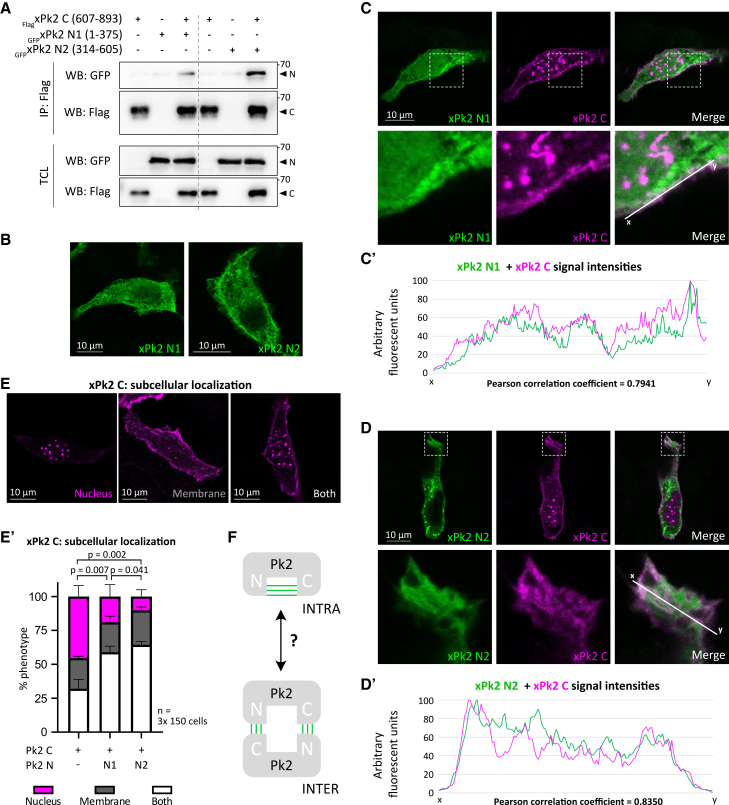
Prickle2 N-terminus interacts with its C-terminus to regulate its subcellular localization (A) Co-immunoprecipitation (IP) of Flag- and GFP-tagged Prickle2 constructs in HEK293 cells. The N-terminal and central fragments (xPk2 N1 and xPk2 N2) efficiently associate with the C-terminal fragment (xPk2 C), as shown by Flag pull-down. (B) Subcellular localization of xPk2 N-terminal fragments (N1 and N2) in HEK293 cells. (C) Co-localization of xPk2 N1 with the Pk2 C-terminal fragment in HEK293 cells. (C′) Line-scan quantification showing overlapping signal intensities along the indicated axis. (D) Co-localization of xPk2 N2 with the Pk2 C-terminal fragment in HEK293 cells. (D′) Line-scan quantification confirming signal overlap. (E) Representative confocal images showing subcellular localization of the Pk2 C-terminal fragment (magenta) in HEK293 cells. When expressed alone, Pk2 C localizes predominantly to the nucleus, whereas co-expression with N1 or N2 fragments promotes redistribution toward the plasma membrane. Scale bars, 10 μm. (E′) Co-expression with N-terminal fragments significantly increases the proportion of cells exhibiting dual membrane-nuclear localization (“both”). Data are presented as mean ± SD; statistical significance was determined using one-way ANOVA followed by Tukey’s post hoc test. (F) Schematic model illustrating intra- and/or intermolecular interactions between the N- and C-terminal regions of Prickle2.

Given that full-length Prickle2 binds Ptprz1b less efficiently than several deletion variants (Figures 1C–1G), we hypothesized that Prickle2 adopts a conformation mediated by an interaction between its N- and C-terminal regions. To test this hypothesis, we performed co-immunoprecipitation assays by using separately expressed FLAG-tagged C-terminal and GFP-tagged N-terminal fragments of *Xenopus* Prickle2 (Figure 2A). Both N-terminal fragments (Pk2 N1 and Pk2 N2) efficiently co-precipitated with the C-terminal region, supporting an N- and C-terminal interaction.

We next examined whether this interaction influences subcellular localization. In HEK293 cells, the isolated Pk2 C-terminal fragment localized predominantly to the nucleus, consistent with the presence of nuclear localization signals (Figures 2B–2E). However, upon co-expression with N-terminal fragments, the C terminus was redistributed toward the cytoplasm and plasma membrane, as demonstrated by increased co-localization with membrane-associated N-terminal constructs (Figures 2C and 2D). Line-scan analyses further confirmed overlapping signal intensities, supporting the physical proximity of these fragments in non-nuclear compartments (Figures 2C′ and 2D′). Quantification of subcellular distribution revealed a clear shift from predominantly nuclear localization of the C-terminal fragment toward cytoplasmic and membrane-associated pools upon co-expression with N-terminal regions, with a marked increase in the “both” category (Figure 2E and 2E′).

Together, these data support a model in which the N-terminal region of Prickle2 interacts with its C terminus to regulate its subcellular distribution. However, based on these experiments, we cannot distinguish whether this interaction occurs intramolecularly within a single Prickle2 molecule or intermolecularly between Prickle2 molecules, and both possibilities remain plausible (Figure 2F).

### VANGL is required for Prickle-Ptprz1b complex formation

**Figure 3:**
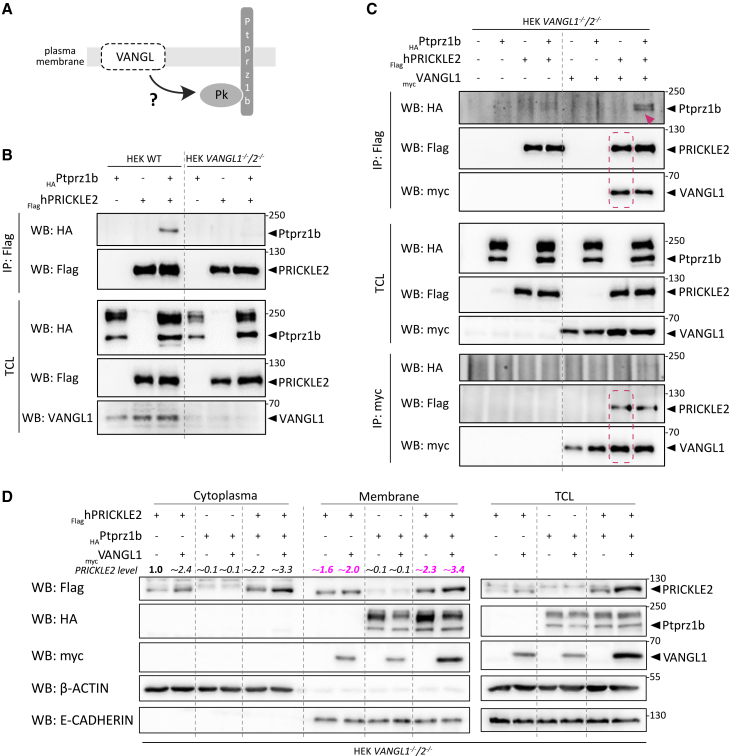
VANGL is required for Prickle-Ptprz1b complex formation (A) Schematic illustration of the experimental strategy to test whether VANGL contributes to the Prickle-Ptprz1b interaction. (B) Co-immunoprecipitation of Ptprz1b with PRICKLE2 in wild-type and VANGL1/2 double-knockout HEK293 cells. The interaction is lost in the absence of VANGL proteins. (C) Immunoprecipitation experiment showing restoration of PRICKLE2-Ptprz1b binding upon re-expression of VANGL1 (marked by the magenta arrow) in VANGL1/2 double-knockout cells. The PRICKLE2-VANGL1 interaction itself is not affected, as indicated by the dotted magenta box. (D) Subcellular fractionation of VANGL1/2 double-knockout HEK293 cells showing that PRICKLE2 is capable of partial membrane association even in the absence of VANGL1/2 and Ptprz1b. Approximately 50% of PRICKLE2 remains cytoplasmic, while ∼50% is associated with the membrane under basal conditions. Co-expression of VANGL increases membrane localization, which is further enhanced by Ptprz1b. Relative PRICKLE2 levels are normalized to the cytoplasmic fraction of PRICKLE2 expressed alone.

Next, we asked whether the presence of VANGL proteins influences the interaction between PRICKLE2 and Ptprz1b (Figure 3A). In HEK293 cells lacking both endogenous VANGL1 and VANGL2,^38^ the interaction between PRICKLE2 and Ptprz1b was not detected, as shown by immunoprecipitation (Figure 3B). When we reintroduced exogenous VANGL1, the binding of Ptprz1b to PRICKLE2 was restored (Figure 3C, magenta arrow), indicating that VANGL is necessary and indispensable for this interaction to occur. Importantly, the interaction between PRICKLE2 and VANGL1 was not affected by the absence of Ptprz1b, consistent with the lack of endogenous PTPRZ1 expression in HEK293 cells (Figure 3C, dashed magenta boxes), and VANGL on its own was not able to bind Ptprz1b, even in the presence of PRICKLE2 (Figure 3C, IP: myc). This points to a model in which VANGL indirectly enables PRICKLE2 to bind Ptprz1b.

Thus, we suggest that Ptprz1b does not interact with VANGL directly but depends on the presence of VANGL in order to engage PRICKLE2 at the plasma membrane. To test this more directly, we performed cell fractionation in HEK293 *VANGL1/2* double-knockout cells. In the absence of VANGL1/2, PRICKLE2 was still weakly detected in the membrane fraction, while the reintroduction of VANGL1 increased PRICKLE2 membrane recruitment. This effect was most pronounced when PRICKLE2 was co-expressed with both VANGL1 and Ptprz1b (Figure 3D). Interestingly, under all conditions, approximately 50% of the PRICKLE2 pool remained associated with the membrane fraction, suggesting that PRICKLE2 has an intrinsic membrane-associated pool that is further stabilized by VANGL and Ptprz1b. In addition, subcellular fractionation revealed that smaller pools of PRICKLE2 were also present in mitochondria and nuclei (Figure S3A).

Together, these data support a model in which VANGL does not simply bind Ptprz1b directly but rather promotes the formation of a membrane-competent PRICKLE2 pool that allows productive Ptprz1b-PRICKLE2 complex formation at the plasma membrane.

### Absence of Ptprz1b leads to Prickle dissociation from the membrane *in vivo*

**Figure 4:**
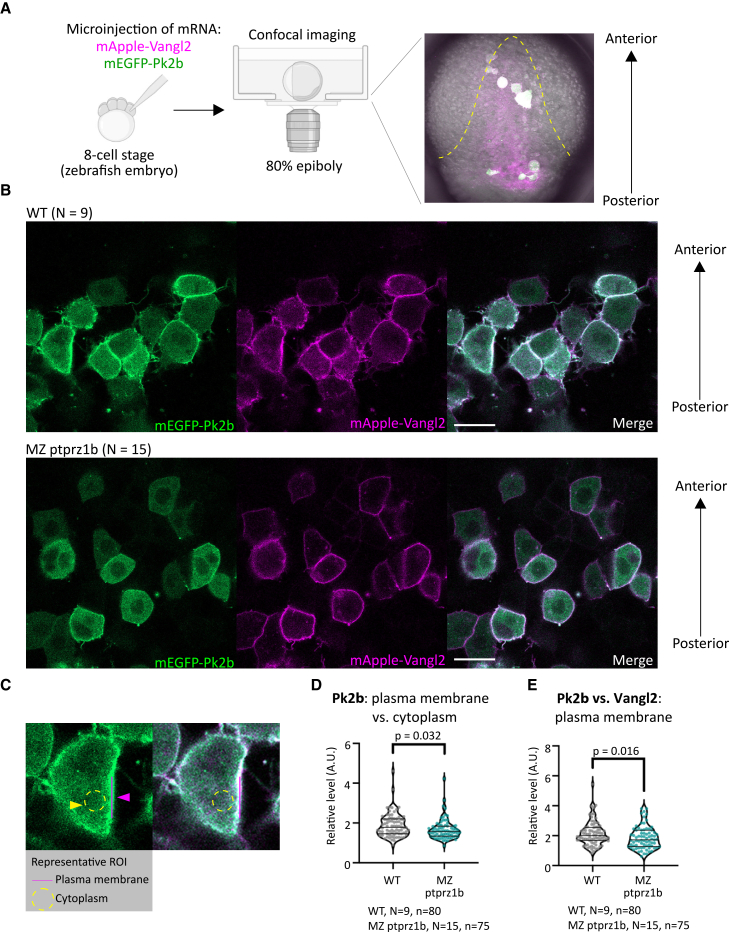
Absence of Ptprz1b leads to Prickle dissociation from the membrane *in vivo* (A) Schematic illustration of the experimental setup. Wild-type (WT) or maternal-zygotic (MZ) *ptprz1b* zebrafish embryos were injected at the 8-cell stage with 100 pg zebrafish *mEGFP-prickle2b* (mEGFP-Pk2b) mRNA and 40 pg mApple-Vangl2 mRNA into a single dorsal blastomere. Embryos were dorsally mounted and imaged at 80% epiboly. Representative bright-field images of WT and MZ *ptprz1b* embryos are shown. (B) Representative confocal single-plane images of cells co-expressing mEGFP-Pk2b and mApple-Vangl2 in WT and MZ *ptprz1b* embryos obtained from three independent experiments. Imaging was performed at comparable Z-depths counting from the enveloping epithelium. Scale bars, 20 μm. (C) Representative region of interest (ROI) used for quantification in (D) and (E). mEGFP-Pk2b and mApple-Vangl2 membrane intensities were measured along a defined membrane segment (magenta line), and mEGFP-Pk2b cytoplasmic intensity was measured in a neighboring circular ROI (yellow dashed circle). (D) Quantification of membrane mEGFP-Pk2b signal normalized to corresponding cytoplasmic signal. MZ *ptprz1b* embryos (*N* = 15, *n* = 75) exhibited a lower membrane-to-cytoplasmic ratio compared with WT (*N* = 9, *n* = 80). Violin plots display the minimum, 25th percentile, median, 75th percentile, and maximum values of the dataset. Individual observations are shown as dots. Mann-Whitney test was performed to calculate *p* value under a confidence interval of 95% (CI = 95%). An asterisk is indicated for comparison showing statistical significance. The number of analyzed embryos and cells is indicated as *N* and *n*, respectively. (E) Quantification of membrane-localized mEGFP-Pk2b signal normalized to mApple-Vangl2 signal. Reduced Pk2b membrane recruitment is observed in MZ *ptprz1b* embryos despite unchanged Vangl2 levels. Violin plots display the minimum, 25th percentile, median, 75th percentile, and maximum values of the dataset. Individual observations are shown as dots. Mann-Whitney test with 95% CI was performed.

To examine the relevance of these interactions in a physiological context, we performed *in vivo* imaging in zebrafish embryos (Figure 4A). In wild-type (WT) embryos co-expressing mEGFP-tagged zebrafish Prickle2b (mEGFP-Pk2b) and mApple-tagged zebrafish Vangl2 (mApple-Vangl2), Prickle2b localized efficiently to the plasma membrane (Figure 4B). In contrast, in maternal-zygotic (MZ) *ptprz1b* mutants absent in *ptprz1b* expression, Prickle membrane localization was significantly reduced, despite normal Vangl2 levels, and cytoplasmic levels were increased (Figure 4B).

Quantitative analysis confirmed that mEGFP-Pk2b showed increased cytoplasm and reduced membrane localization in the absence of endogenous Ptprz1b (Figures 4B–4E). Vangl2 localization remained unchanged, reinforcing the idea that Ptprz1b specifically affects Prickle2b positioning without broadly disrupting PCP components (Figure S3B). Together, these results demonstrate that Ptprz1b is required for efficient membrane recruitment of Prickle *in vivo*, providing a mechanistic link between Ptprz1b activity and Prickle subcellular localization.

### Endogenous Ptprz1 deficiency leads to PCP defects in zebrafish and *Xenopus*

**Figure 5:**
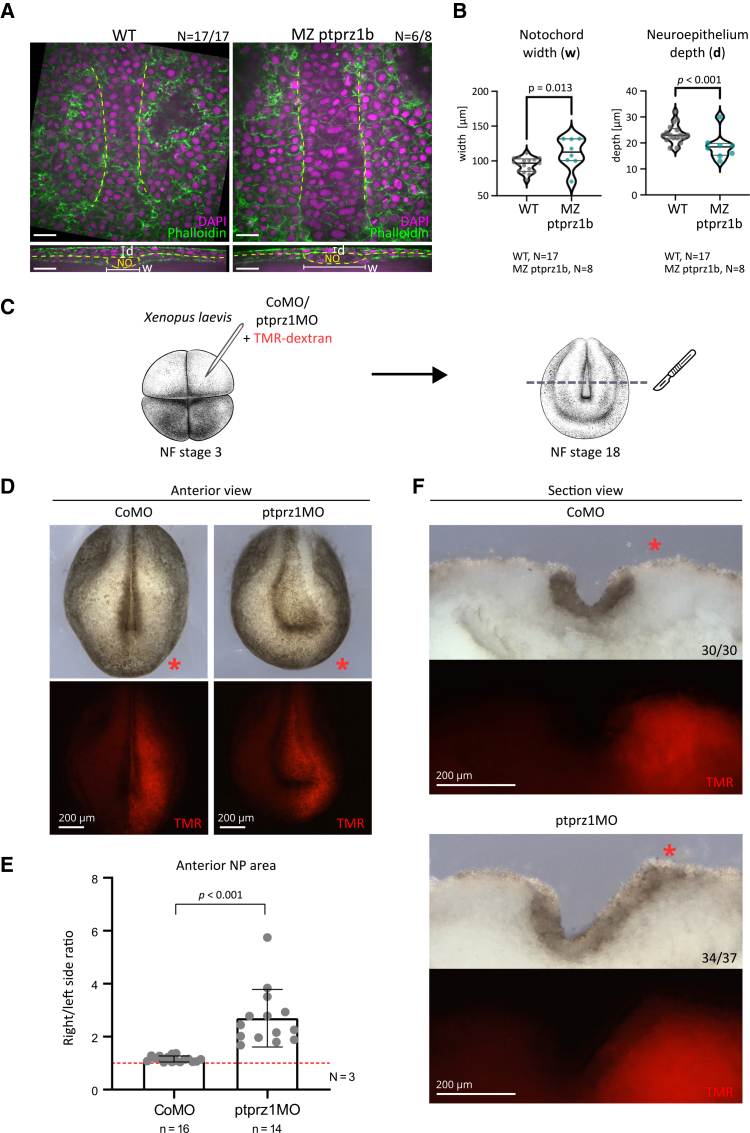
Endogenous Ptprz1 deficiency leads to PCP defects in zebrafish and *Xenopus* (A) Phalloidin (green) and DAPI (magenta) staining of WT and MZ *ptprz1b* mutants at 10 hpf. Single plane images at the notochord with maximal width are shown at the top, and reconstructed cross-sectional views at the bottom. 6 out of 8 MZ *ptprz1b* mutants displayed broadened notochord and thinner neuroepithelium when compared with WT embryos (*N* = 17). Dotted yellow lines in top images label the edge of notochord, while those in bottom images delineate the border between neuroepithelium and mesoderm and outline the notochord structure. White lines indicate the corresponding measurements of maximal width of notochord and depth of neuroepithelium for statistical analyses in (B). Scale bars, 50 μm. (B) Quantification and statistical analyses on maximal width of notochord and depth of neuroepithelium in WT (*N* = 17) and MZ *ptprz1b* mutants (*N* = 8). Violin plots display the minimum, 25th percentile, median, 75th percentile, and maximum values of the dataset. Individual observations are shown as dots. Statistical analyses were performed on Estimation Stats (www.estimationstats.com) with a confidence interval (CI) of 95%. The calculated *p* values are labeled, and asterisks are indicated for comparison showing statistical significance (*p* < 0.05, CI = 95%). (C) Schematic of microinjection strategy of control morpholino (CoMO) or *ptprz1* morpholino (*ptprz1* MO) in *Xenopus* embryos. One dorsal blastomere at the animal pole of 4-cell stage embryos was injected to achieve unilateral targeting. (D) Representative NF stage 18 embryos. Bright-field images (top) show defects in anterior neural tube (NT) closure on the *ptprz1* MO-injected side. The injected side is identified by the TMR-dextran tracer (bottom). Scale bars, 200 μm. (E) Quantification of anterior neural plate (NP) area, shown as the ratio between right and left sides. *ptprz1* MO-injected embryos exhibit significant defects in anterior NT closure compared with controls. The dashed red line represents the average value of CoMO condition; (*p* < 0.001). Values are presented as mean ± SD. Statistical significance was determined using an unpaired Student’s *t* test. (F) Transverse sections of NF stage 18 embryos at the level of the anterior NT. Views of cut surfaces reveal abnormal neural fold morphology on the injected side (red asterisk). Numbers in the bottom right corner indicate the proportion of embryos exhibiting the representative phenotype shown in each panel relative to the total analyzed. Scale bars, 200 μm.

To assess the *in vivo* function of Ptprz1 in PCP-regulated morphogenetic processes, we first analyzed MZ *ptprz1b* mutant zebrafish embryos at 10 hours post-fertilization (hpf), the endpoint of gastrulation. Phalloidin and DAPI staining revealed clear defects in axial organization, with most *ptprz1b* mutants (*N* = 6/8) displaying a broadened notochord and a concomitantly reduced neuroepithelium thickness compared with WT embryos (Figure 5A). Quantification confirmed a significant increase in notochord width together with a decrease in neuroepithelium depth (Figure 5B), consistent with impaired convergent extension movements, i.e., a hallmark PCP readout.

To determine whether Ptprz1 plays a similar role in another vertebrate model, we performed unilateral knockdown of *ptprz1* in *Xenopus* embryos. Targeted injection of *ptprz1* morpholino oligos into one dorsal blastomere at the 4-cell stage resulted in asymmetric closure defects in anterior neural tube at Nieuwkoop and Faber (NF) stage 18 (Figures 5C and 5D). The injected side, identified by tetramethylrhodamine (TMR)-dextran tracer, exhibited clear abnormalities in neural fold convergence compared with the uninjected side.

Quantitative analysis demonstrated a significant increase in the ratio between right and left anterior neural plate areas, indicating defective neural plate folding upon Ptprz1 depletion (Figure 5E). Consistently, transverse sections through the anterior neural tube revealed disrupted neural fold morphology on the injected side, further confirming disturbed neurulation (Figure 5F).

Together, these results demonstrate that endogenous Ptprz1 is required for proper PCP-dependent morphogenesis across vertebrates, regulating convergent extension in zebrafish and neural plate folding in *Xenopus*.

### *Drosophila* Ptprz1 homolog does not affect invertebrate PCP, whereas *Drosophila* Prickle recapitulates vertebrate Prickle function in zebrafish

To test whether the observed PCP phenotypes are conserved across vertebrate and invertebrate species, we next turned to *Drosophila*, where PCP signaling has both conserved and distinct features compared with vertebrates. In flies, Midkine-PTPRZ1 signaling is conserved in the form of orthologs Miple and PTP99A, respectively.^39^^,^^40^ Although PTP99A has been implicated in polarity-related processes such as ommatidial rotation and collective cell migration,^41^^,^^42^ its role in classical PCP remains unclear.

**Figure 6:**
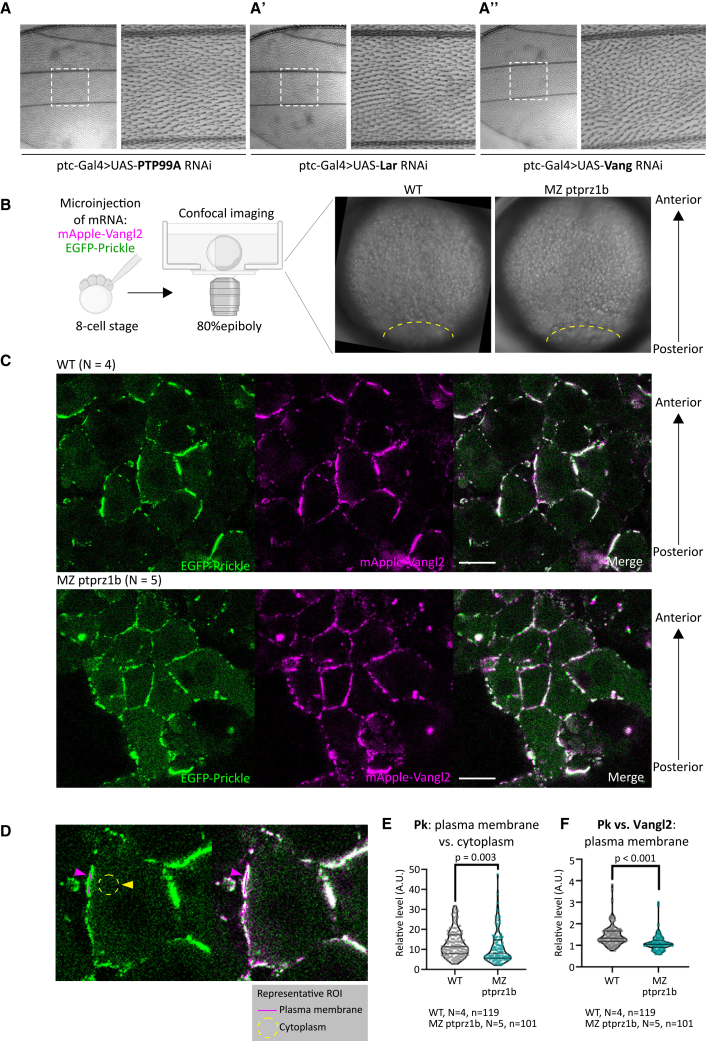
*Drosophila* Ptprz1 homolog does not affect invertebrate PCP, whereas *Drosophila* Prickle recapitulates vertebrate Prickle function in zebrafish (A) *Drosophila* wing images. Patched-Gal4 was used to drive RNAi-mediated knockdown of PTP99A (A), Lar (A′), and Vang (A″) in the anterior compartment of the wing. Images on the right show magnified views of the indicated regions. While Vang knockdown disrupts wing hair polarity, knockdown of PTP99A or Lar does not affect polarity, indicating that these PTPs are not required for classical PCP in this context. (B) Schematic illustration of the experimental setup. WT or MZ *ptprz1b* zebrafish embryos were injected at the 8-cell stage with 100 pg *Drosophila EGFP-prickle* (*EGFP-Pk*) mRNA and 40 pg *mApple-vangl2* mRNA into a single cell of dorsal blastomere. Embryos were dorsally mounted and imaged at 80% epiboly, and representative bright-field images of WT and MZ *ptprz1b* embryos are shown. (C) Representative confocal single-plane images of cells at comparable Z-depths co-expressing EGFP-Pk and mApple-Vangl2 in WT (*N* = 4) and MZ *ptprz1b* (*N* = 5) embryos. The experiment was repeated three times. (D) Representative region of interest (ROI) used for quantification in (E) and (F). Membrane EGFP-Pk and mApple-Vangl2 intensities were measured along a defined plasma membrane segment (magenta line), and cytoplasmic EGFP-Pk intensity was measured in a neighboring circular ROI (yellow dashed circle). (E) Quantification of membrane EGFP-Pk signal normalized to corresponding cytoplasmic signal. MZ *ptprz1b* embryos (*N* = 5, *n* = 101) exhibited a lower membrane-to-cytoplasmic ratio compared with WT (*N* = 4, *n* = 119). Violin plots display the minimum, 25th percentile, median, 75th percentile, and maximum values of the dataset. Individual observations are shown as dots. Mann-Whitney test with 95% CI was performed (∗*p* < 0.05). N indicates number of embryos analyzed, and n number of cells. (F) Quantification of membrane-localized EGFP-Pk signal normalized to mApple-Vangl2 signal. Reduced Prickle membrane recruitment is observed in MZ *ptprz1b* embryos despite unchanged Vangl2 levels after Mann-Whitney test (∗*p* < 0.05; CI = 95%). Violin plots display the minimum, 25th percentile, median, 75th percentile, and maximum values of the dataset. Individual observations are shown as dots.

To address this, we analyzed wing hair orientation following RNAi-mediated knockdown of PTP99A and its close homolog Lar. In contrast to Vang (*Drosophila* homolog of vertebrate Vangl) knockdown, which strongly disrupted wing hair polarity, knockdown of PTP99A or Lar did not affect wing hair alignment (Figure 6A, 6A′, and 6A″). These results indicate that, unlike vertebrate Ptprz1, the *Drosophila* ortholog PTP99A does not play a detectable role in this classical PCP output, suggesting divergence in the upstream regulation of PCP between invertebrates and vertebrates.

We next asked whether the function of Prickle itself is conserved. To this end, we expressed *Drosophila* Prickle in zebrafish embryos and analyzed its localization and interaction with PCP components. Strikingly, *Drosophila* Prickle (EGFP-Prickle) recapitulated the behavior of vertebrate Prickle2, showing membrane enrichment and co-localization with Vangl2 at cell-cell junctions in WT embryos (Figures 6B and 6C). This membrane association was reduced in MZ *ptprz1b* mutants, indicating that, similar to vertebrate Prickle2, *Drosophila* Prickle depends on Ptprz1 for efficient membrane retention.

Quantitative analysis confirmed a significant reduction in the membrane-to-cytoplasm ratio of Prickle signal in *ptprz1b* mutants (Figure 6E), as well as decreased co-localization with Vangl2 (Figure 6F). The localization of Vangl2 itself was not affected under these conditions (Figure S5A). These data demonstrate that while the upstream receptor Ptprz1 function is not conserved in *Drosophila* PCP, the molecular behavior of Prickle is evolutionarily conserved and remains dependent on Ptprz1 in the vertebrate context.

Together, these findings reveal a divergence at the level of PCP regulation, where the receptor function of Ptprz1 is vertebrate-specific, whereas the downstream role of Prickle in membrane-associated PCP complexes is conserved across species.

### Ptprz1b promotes Prickle retention at the plasma membrane in zebrafish EVL cells

Since Ptprz1b is required for Prickle membrane recruitment *in vivo*, we next asked whether it also contributes to Prickle’s plasma membrane stability during epithelial polarization. In early zebrafish embryos, immature epidermal cells that constitute the enveloping cell layer (EVL) already exhibit epithelial polarity.^43^ They have established apicobasal polarity on the plasma membrane with their apical side facing the external environment and basal side oriented toward the deep cell layer beneath the EVL. Interestingly, overexpressed *Drosophila* Dendra2-Pk displayed polarized localization as multiple puncta on both apical and basal sides of the EVL cells (Figure S4). This provides a model to investigate whether Ptprz1b stabilizes Prickle at the plasma membrane during establishment of epithelial cell polarity.

**Figure 7:**
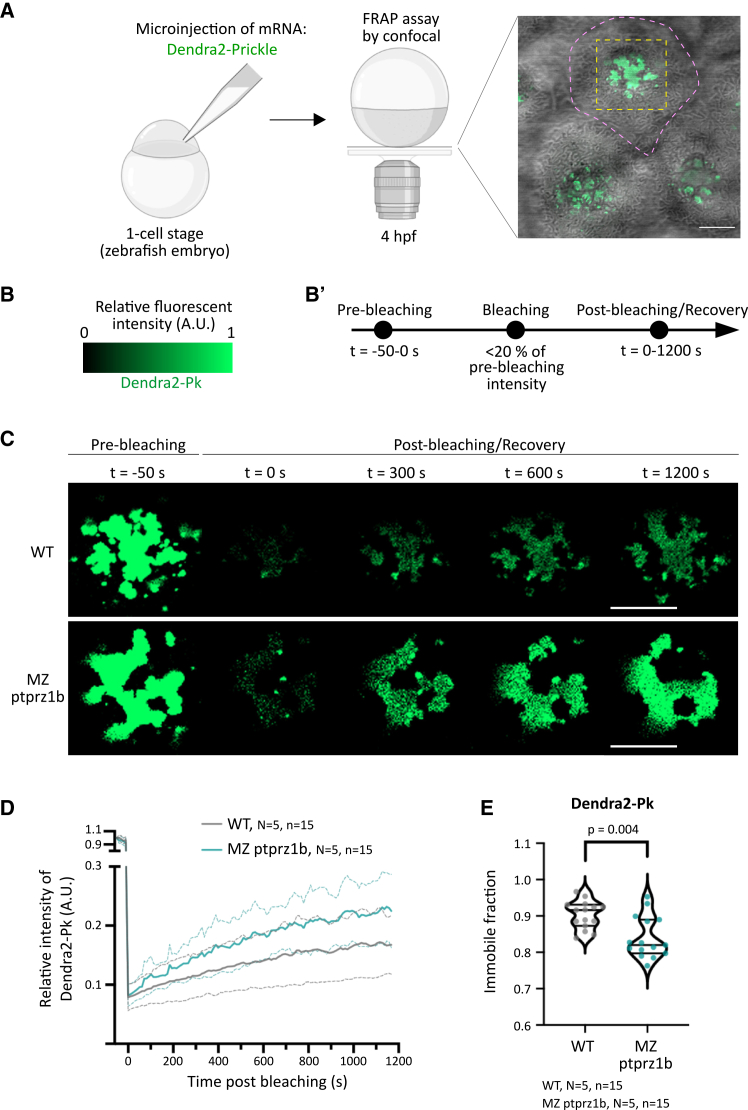
Ptprz1b promotes Prickle retention at plasma membrane in zebrafish EVL cells (A) Illustration of the experimental setup (left two images). WT or MZ *ptprz1b* embryos were co-injected with 100 pg of *Dendra2-prickle* (*Dendra2-pk*) mRNA at the 1-cell stage. Injected embryos were mounted with the animal pole facing down and imaged using confocal microscopy at 4 hpf. Gray dashed line indicates the Z-plane of acquisition shown in the rightmost image. The rightmost image shows representative DIC and fluorescent image of the apical cortex of EVL cells in WT embryos. Magenta dashed line delineates the EVL cell, and yellow dashed square indicates the region of interest (ROI) for FRAP measurement. Scale bars, 10 μm. (B) Schematic FRAP timeline of pre-bleaching acquisition, photobleaching, and post-bleaching acquisition during recovery. (C) Representative FRAP series of Dendra2-Pk within the ROI of WT (*N* = 5) and MZ *ptprz1b* embryos (*N* = 5) obtained from three repeats. Pre-bleaching and post-bleaching/recovery images are pseudo-colored based on the fluorescent intensities of Dendra2-Pk relative to the first pre-bleaching timepoint. Scale bars, 10 μm. (D) Normalized relative fluorescent intensity FRAP curves of Dendra2-Pk acquired from WT (*N* = 5, *n* = 15) and MZ *ptprz1b* embryos (*N* = 5, *n* = 15). Solid lines show the mean of relative fluorescent intensity, while dashed lines indicate the upper and lower standard deviation (±SD). The number of analyzed embryos and cells is shown as *N* and *n*, respectively. (E) Immobile fraction of Dendra2-Pk calculated from the FRAP experiment. Violin plots display the minimum, 25th percentile, median, 75th percentile, and maximum values of the dataset. Individual observations are shown as dots. Statistical analyses were performed using Mann-Whitney test with a CI of 95%. Calculated *p* value is labeled for each comparison, and an asterisk is used to show statistical significance (*p* < 0.05).

We conducted FRAP (fluorescence recovery after photobleaching) experiments on the apical cortex of EVL cells overexpressing *Drosophila* Dendra2-Pk in WT and MZ *ptprz1b* embryos at 4 hpf (Figure 7A). After photobleaching, the fluorescence of Dendra2-Pk recovered significantly faster in MZ *ptprz1b* embryos compared with WT, indicating an increase in Pk turnover when *ptprz1b* was mutated (Figures 7B–7D, Video S1). Consistently, we observed a significant decrease in the immobile fraction of Dendra2-Pk in MZ *ptprz1b* embryos in comparison to WT (Figure 7E). These results highlight the role of Ptpz1b in promoting Prickle retention at the plasma membrane during the establishment of polarity in EVL cells.

Video S1. Time-lapse of Dendra2-Prickle fluorescence during FRAP in WT and MZ ptprz1b embryos, corresponding to the experiment shown in Figure 7 After photobleaching, WT cells show limited and slower fluorescence recovery, whereas MZ ptprz1b mutants display faster and more extensive recovery, consistent with reduced membrane retention of Prickle in the absence of Ptprz1b. Scale bar, 10 μm.

### Ptprz1b phosphatase activity modulates Prickle binding and membrane retention

**Figure 8:**
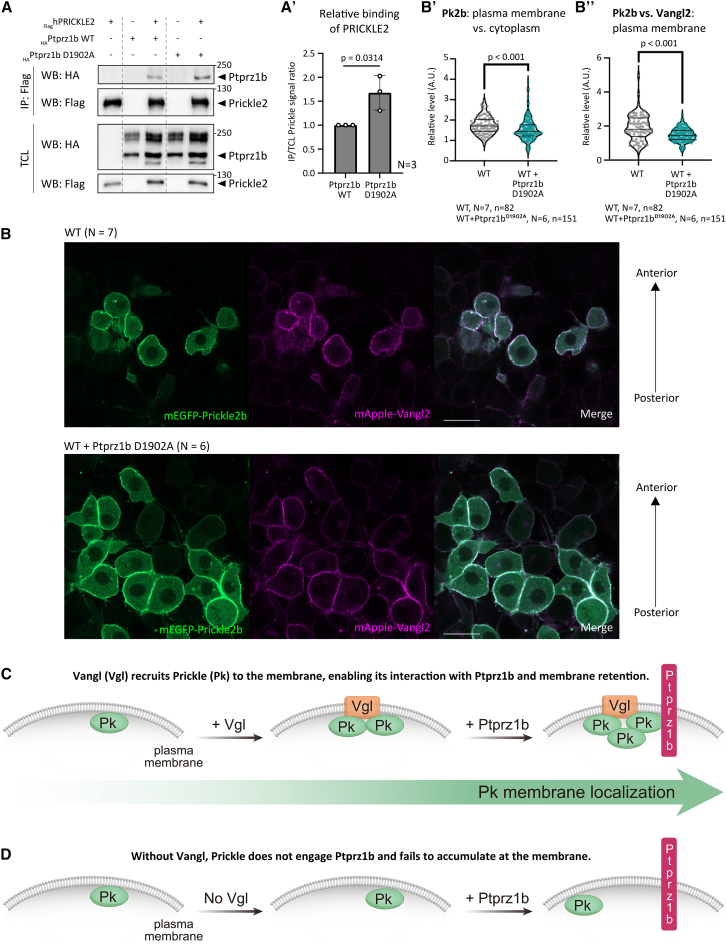
Ptprz1b phosphatase activity modulates Prickle binding and membrane retention (A) Co-immunoprecipitation of PRICKLE2 with WT or substrate-trap Ptprz1b^D1902A^. The D1902A mutant shows increased binding to PRICKLE2 compared with WT. (A′) Quantification of Ptprz1b binding levels. Data are presented as mean ± SD. Statistical significance was determined using an unpaired two-tailed Student’s *t* test. (B) Representative images of mEGFP-Prickle2b (mEGFP-Pk2b) and mApple-Vangl2 localization in WT embryos with (*N* = 6) or without Ptprz1b^D1902A^ (*N* = 7) from three independent repeats. (B′) Quantification of mEGFP-Pk2b membrane vs. cytoplasm localization. The addition of Ptprz1b^D1902A^ mutant in WT (*N* = 6, *n* = 151) reduces Prickle membrane retention compared with WT without Ptprz1b^D1902A^ (*N* = 7, *n* = 82). Mann-Whitney test was performed under 95% CI (∗*p* < 0.05). Violin plots display the minimum, 25th percentile, median, 75th percentile, and maximum values of the dataset. Individual observations are shown as dots. The number of embryos and cells is labeled as *N* and *n*, respectively. (B″) Quantification of mEGFP-Pk2b vs. Vangl2 membrane localization. The addition of Ptprz1b^D1902A^ mutant in WT (*N* = 6, *n* = 151) reduces their membrane retention compared with WT without Ptprz1b^D1902A^ (*N* = 7, *n* = 82). Mann-Whitney test was performed under 95% CI (∗*p* < 0.05). Violin plots display the minimum, 25th percentile, median, 75th percentile, and maximum values of the dataset. Individual observations are shown as dots. The number of embryos and cells is labeled as *N* and *n*, respectively. (C) Proposed model: Vangl (Vgl) recruits Prickle (Pk) to the membrane, enabling interaction with Ptprz1b, which promotes membrane retention in an activity-dependent manner. (D) Proposed model: in the absence of Vangl (No Vgl), Prickle (Pk) fails to engage Ptprz1b and does not accumulate at the membrane.

To further dissect the role of Ptprz1b enzymatic activity in Prickle regulation, we employed a substrate-trapping mutant of Ptprz1b (D1902A),^44^ which was reported catalytically inactive but exhibits enhanced binding to its substrates. Co-immunoprecipitation assays revealed that PRICKLE2 binds more strongly to the Ptprz1b D1902A mutant compared with WT embryos (Figure 8A–A′), consistent with stabilization of a substrate-enzyme complex.

We next examined the functional consequence of this strengthened interaction *in vivo*. Quantitative analysis of Prickle2b localization showed that zebrafish WT embryos expressing the Ptprz1b D1902A variant displayed reduced membrane retention of Prickle2b when compared with WT embryos (Figure 8B–B″). The localization of Vangl2 itself was not affected under these conditions (Figure S5A). Thus, while the addition of Ptprz1b trapping mutant enhances binding, it impairs proper membrane stabilization of Prickle2b, indicating that Ptprz1b catalytic activity is required for efficient Prickle2b retention at the plasma membrane.

Based on these findings, we propose a model in which Vangl first recruits Prickle2 to the plasma membrane, enabling its interaction with Ptprz1b (Figure 8C). In this context, Ptprz1b promotes stable membrane retention of Prickle2, dependent on its phosphatase activity. In the absence of Vangl, Prickle2 fails to efficiently associate with Ptprz1b and does not accumulate at the membrane (Figure 8D). Together, these data support a sequential mechanism in which Vangl establishes a membrane-competent pool of Prickle2, while Ptprz1b reinforces its retention through activity-dependent regulation.

## Discussion

Our study identifies Ptprz1 as a regulator of Prickle2 membrane retention and reveals a previously unrecognized mechanism in which Ptprz1b promotes membrane-associated Prickle2 stability through VANGL-dependent and phosphatase activity-dependent regulation (Figure 8). These findings position Ptprz1b as a previously unrecognized and functionally relevant *in vivo* modulator of the Wnt/PCP pathway.

### Prickle2 interacts with Ptprz1b via three distinct regions

Through systematic truncation analysis, we identified that multiple regions of Prickle2, including the LIM domain-containing N terminus, central region, and C terminus, contribute to Ptprz1b binding. LIM domains are well-known scaffolds mediating protein-protein interactions within polarity, cytoskeletal, and transcriptional networks.^45^ Notably, several deletion constructs showed a stronger association with Ptprz1b compared with full-length Prickle2, suggesting that the full-length protein may adopt a conformation in which binding sites are partially masked.

Consistent with this model, we observed an interaction between the N- and C-terminal regions of Prickle2, which correlated with changes in the subcellular localization of the C-terminal fragment. However, our data do not distinguish whether this interaction occurs intra- or intermolecularly. These observations are compatible with a model in which conformational regulation of Prickle2 influences its accessibility to Ptprz1b and its subcellular distribution. This is in line with previous work showing that the PET and LIM domains of Prickle cooperate to regulate membrane association and the protein’s structural properties.^46^

### VANGL enables Ptprz1b-Prickle complex formation

Importantly, we show that VANGL1 protein is required for Ptprz1b to associate with Prickle2. VANGL proteins are the best characterized interactors of PRICKLE proteins to date.^22^^,^^47^^,^^48^^,^^49^^,^^50^^,^^51^^,^^52^^,^^53^^,^^54^^,^^55^^,^^56^^,^^57^^,^^58^^,^^59^^,^^60^^,^^61^^,^^62^ In the absence of VANGL1/2, the Ptprz1b-Prickle2 interaction is reduced. This is consistent with a model in which VANGL may act as a structural organizer or scaffold for higher-order PCP complexes, possibly by stabilizing Prickle conformations competent for Ptprz1b binding. However, our data do not clearly distinguish whether VANGL acts as a direct structural component of a ternary Ptprz1b-VANGL/Prickle complex, or whether it indirectly enables the interaction by promoting a membrane-competent pool of Prickle.

Our data align with previous studies demonstrating that VANGL can control Prickle2 protein stability via polyubiquitination^55^ and that its phosphorylation and membrane localization are tightly regulated by Wnt5a and CK1.^25^^,^^26^ However, unlike VANGL’s controversial role in targeting Prickle for degradation,^55^ our findings suggest that VANGL also enables Prickle membrane recruitment when complexed with Ptprz1b, pointing to a dual role in both turnover and membrane anchoring.

That VANGL is essential for Ptprz1b-Prickle complex formation but not directly bound by Ptprz1b (Figure S3) suggests a coordinated assembly mechanism. Such an arrangement is reminiscent of ternary complexes in other Wnt-related modules, including Fz/Dvl-related plasma membrane complexes,^15^^,^^16^^,^^17^^,^^18^^,^^19^^,^^20^^,^^21^ where indirect bridging ensures signal fidelity and spatial control. We note, however, that the loss of interaction in VANGL1/2-deficient cells could reflect reduced membrane availability of Prickle rather than a strict requirement for VANGL in the physical association *per se*. Testing membrane-tethered Prickle variants will help discriminate between these alternatives.

### Ptprz1b regulates prickle localization and turnover *in vivo*

Importantly, we validated the physiological relevance of these interactions in zebrafish embryos, where loss of Ptprz1b severely compromises Prickle membrane localization while sparing Vangl2. This uncovers a previously unknown *in vivo* function for Ptprz1b in stabilizing the membrane-associated Prickle pool.

Consistent with this, FRAP analysis in zebrafish EVL cells revealed a markedly faster turnover of Prickle in *Ptprz1b* mutants compared with WT, accompanied by a reduced immobile fraction. These dynamic measurements indicate that Ptprz1b promotes Prickle retention at the plasma membrane during the establishment of epithelial polarity, supporting its role as a stabilizing anchor. Together, these *in vivo* findings establish Ptprz1b as a key determinant of Prickle membrane recruitment and stabilization. Given the increasing appreciation for the dynamic membrane trafficking of Wnt/PCP components, including endocytosis via clathrin-coated pits and internalization via syndecans and protocadherins,^63^^,^^64^ it is conceivable that Ptprz1b, a receptor-type phosphatase enriched at the plasma membrane, anchors Prickle in a way that counters these removal processes.

Moreover, Ptprz1b′s involvement may provide a mechanistic explanation for how cells spatially restrict Prickle to polarized cortical domains. This function may be particularly critical in rapidly remodeling tissues during development and may extend to cancer cell migration and invasion, where altered PCP signaling contributes to metastasis.^13^ Misregulation of Wnt pathway modulators, including VANGL, R-spondins, RNF43/ZNRF3, and GPI-anchored proteins, is frequent in aggressive tumors of the lung, colon, and liver, implicating membrane composition and receptor complex dynamics as promising therapeutic targets.^11^^,^^12^^,^^13^^,^^14^ In line with this notion, PTPRZ1 is one of the top upregulated genes in metastatic glioma and has been shown to activate Rho/ROCK signaling to migrate along a gradient of its ligand, pleiotrophin (PTN).^35^ We, therefore, hypothesize that secreted growth factors (e.g., PTN) may modulate PTPRZ1-dependent stabilization of PRICKLE at the membrane; direct ligand-dependence has yet to be demonstrated.

### Divergent role of Ptprz1 in vertebrate and invertebrate PCP, with conserved Prickle function

Our comparative analysis across species further suggests that Ptprz1-mediated regulation of PCP is not fully conserved. While loss of Ptprz1 function resulted in clear PCP-associated phenotypes in both zebrafish and *Xenopus*, including defects in convergent extension and neural plate morphogenesis, depletion of the *Drosophila* homolog PTP99A did not affect classical PCP readouts in the fly wing. This indicates that, although Ptprz1 contributes to PCP-dependent morphogenesis in vertebrates, its role is not conserved in invertebrates for all PCP outputs. Notably, despite this divergence in upstream regulation, *Drosophila* Prickle retained functional compatibility in the vertebrate context, as its expression in zebrafish recapitulated the membrane localization and Vangl2 co-association observed for vertebrate Prickle2, and remained sensitive to Ptprz1b loss. Together, these findings support a model in which the core molecular behavior of Prickle is evolutionarily conserved, whereas the regulatory input from Ptprz1 represents a vertebrate-specific layer that modulates the stability of the PCP complex at the plasma membrane.

### Potential relevance of the PTPRZ1-Prickle/VANGL axis beyond development

Our findings uncover a possible association between PTPRZ1 and PRICKLE/VANGL observed through live imaging in zebrafish embryos. Unlike Ryk, a pseudokinase Wnt receptor, or Rack1, a scaffolding adaptor, PTPRZ1 is a receptor-type tyrosine phosphatase with a well-documented role in glioma and other cancers, raising the possibility that this interaction may be relevant in cancer-related contexts. Given PTPRZ1’s enriched expression in glioblastoma and other invasive cancers, this interaction may support pathological behaviors such as enhanced cell migration or disrupted neuronal development.^36^ However, whether this mechanism contributes to pathological cell migration or cancer progression remains to be tested directly.

### A new view of Prickle as an active membrane hub

Traditionally, Prickle proteins have been viewed as downstream effectors or passive carriers within PCP networks, particularly in contrast to Dvl, which is considered a primary signal transducer.^65^^,^^66^ However, recent studies and our current findings challenge this hierarchy. Emerging roles of Prickle in directing cortical asymmetry, controlling cytoskeletal architecture, and regulating membrane trafficking, e.g.,^37^^,^^67^^,^^68^ suggest that Prickle proteins are not merely substrates but active scaffolds whose positioning and interaction with transmembrane partners such as PTPRZ1 critically shape PCP output.

Together, our findings identify PTPRZ1 as a key regulator of Prickle membrane localization and suggest a new layer of complexity in the spatial organization of the Wnt/PCP signaling machinery. By stabilizing Prickle at the plasma membrane in a VANGL-dependent manner, PTPRZ1 may contribute to the precise tuning of polarity cues in development and disease.

In this context, we propose that while Wnt ligands initiate the asymmetric distribution of Fz/Dvl and Vangl/Prickle complexes, ligand-dependent activation of PTPRZ1 could act to maintain this asymmetry by stabilizing the Vangl/Prickle module at the plasma membrane, although direct ligand dependence remains to be tested experimentally.

### Limitations of the study

While our study identifies Ptprz1b as a regulator of Prickle membrane retention, several limitations remain. First, although our data support a phosphatase activity-dependent mechanism, the direct substrate(s) of Ptprz1b within the PCP pathway remain unknown. Second, while Ptprz1b and Prickle2 robustly associate in biochemical assays, it is not yet clear whether this interaction is direct or mediated by additional membrane-associated components. Third, although our findings are consistent with a model in which extracellular signals may regulate Ptprz1b-dependent stabilization of Prickle, potential ligand dependence, including regulation by PTN, was not directly tested. Finally, a substantial part of the biochemical interaction analysis was performed in HEK293 cells, and therefore, additional studies in endogenous vertebrate tissues will be important to further validate the physiological organization of the Ptprz1b-Prickle complex *in vivo*. Although these questions remain to be addressed, they do not alter the main conclusion that Ptprz1b acts as a physiologically relevant regulator of Prickle membrane localization and PCP signaling in vertebrates.

## Resource availability

### Lead contact

Further information and requests for resources and reagents should be directed to and will be fulfilled by the lead contact, Jakub Harnos (harnos@sci.muni.cz).

### Materials availability

This study generated several plasmid constructs, including HA-ptprz1b D1902A, pCS2+-mApple-vangl2, pCS2+-Dendra2-prickle, pCS2+-mEGFP-prickle2b, and Prickle2 truncation constructs described in the manuscript. All unique/stable reagents generated in this study are available from the lead contact with a completed materials transfer agreement.

### Data and code availability

• All data supporting the findings of this study are included in the article and its supplemental information. Original microscopy images, immunoblot data, and quantitative datasets are available from the lead contact upon reasonable request.
• This study did not generate custom code.
• No large-scale datasets were generated in this study.
• Any additional information required to reanalyze the data reported in this paper is available from the lead contact upon request.

## Acknowledgments

We would like to thank Eva Slabakova and Lenka Doubkova for excellent administrative services. We thank Prof. Vitezslav Bryja for generously providing the HEK293 VANGL1^−/−^/2^−/−^ cell line. We would like to thank Dr. Vendula Hlavackova-Pospichalova for help with subcellular fractionation of tissue culture cells. We gratefully acknowledge support from the Grant Agency of Masaryk University (project no. MUNI/JS/1952/2025) and Czech Ministry of Education, Youth and Sports (project no. CZ.02.01.01/00/22_010/0003229), both awarded to J.H., which enabled this research. Additional support was provided by the Singapore Ministry of Education (project nos. MOE2016-T3-1-005 and MOE-T2EP30221-0008), both awarded to C.W. The funders had no role in study design, data collection and analysis, decision to publish, or preparation of the manuscript.

## Author contributions

Y.L., S.N., L.A.M., and N.S.T. performed experiments and analyzed the data; Y.L. and J.H. drafted the manuscript and performed a literature search; S.N., L.A.M., N.S.T., and C.W. edited the manuscript; C.W. and J.H. conceived the main ideas, secured the funding, and supervised the overall work.

## Declaration of interests

The authors declare no competing interests.

## STAR★Methods

### Key resources table

**Table:** 

REAGENT or RESOURCE	SOURCE	IDENTIFIER
**Antibodies**		

Flag M2 Affinity Gel	Millipore/Merck	A2220, RRID:AB_10063035
GFP B-2	Santa Cruz	sc-9996, RRID:AB_3713277
c-myc 9E10	SC Exbio	sc-40, RRID:AB_62726
HA	Abcam	ab9110, RRID:AB_307019
Flag	Sigma-Aldrich	7425, RRID:AB_439687
GFP	Fitzgerald	20R-GR-011, RRID:AB_1286217
myc	Sigma	C3956, RRID:AB_439680
Flag M2	Sigma/Merck	F1804, RRID:AB_262044
Vangl1 E-3	Santa Cruz	SC-166844, RRID:AB_2212969
pVangl1/2	Abclonal	AP1206
ß-actin	Cell Signaling	4970, RRID:AB_2223172
E-cadherin	Cell Signaling	3195, RRID:AB_2291471
ß-catenin	BD	610153, RRID:AB_397554
HSP60	Cell Signaling	4870, RRID:AB_2295614
Cox IV	Cell Signaling	4850, RRID:AB_2085424
Anti-Mouse IgG Peroxidase antibody	Sigma Aldrich	A4416, RRID:AB_258167
Anti-Rabbit IgG Peroxidase antibody	Sigma Aldrich	A0545, RRID:AB_257896
Alexa Fluor 488 Anti-Mouse	Invitrogen	A32766, RRID:AB_2762823
Alexa Fluor 568 Anti-Rabbit	Invitrogen	A11011, RRID:AB_143157
Cy3 AffiniPure Anti-Rabbit	Jackson ImmunoResearch	711-165-152, RRID:AB_2307443

**Chemicals, peptides, and recombinant proteins**		

Dulbecco’s modified Eagle medium	Gibco	REF41966-029
Fetal bovine serum	Gibco	10270
Protease inhibitor	Roche	11836145001
Phosphatase inhibitor cocktail II	Merck	524625
Protein G sepharose 4 fast flow	GE Healthcare	17-0618-01

**Critical commercial assays**		

mMESSAGE mMACHINE SP6 Transcription Kit	ThermoFisher	AM1340
Qubit™ RNA HS Assay Kit	ThermoFisher	Q32852
2 x Phanta Max Master Mix	Vazyme	P515
2 x Rapid Taq Master Mix	Vazyme	P222
CloneExpress II One Step Cloning Kit	Vazyme	C112
*XhoI*	ThermoFisher	ER0691
*XbaI*	ThermoFisher	ER0681
*EcoRI*	ThermoFisher	ER0271
FastDigest *NotI*	ThermoFisher	FD0593
FastDigest KpnI	ThermoFisher	FD0524
Amersham ECL Prime Luminol Enhancer Solution	Cytiva	29018903
QuikChange II Site-Directed Mutagenesis Kit	Agilent	200523-5

**Experimental models: Cell lines**		

HEK293 WT cells	ATCC	ATCC CRL-1573
HEK293 VANGL1^-/-^/2^-/-^ cells	Mentink et al.^38^	N/A

**Experimental models: Organisms/strains**		

Zebrafish: DBSWT	Department of Biological Sciences, National University of Singapore	N/A
Zebrafish: *ptprz1b*^*-/-*^	Department of Biological Sciences, National University of Singapore	Le et al.^36^
Xenopus laevis WT	European Xenopus Research Centre, Portsmouth	N/A

**Oligonucleotides**		

Primer: *mApple* CE F1: TCGAATTC AAGGCCTCTCGAGGCCACCATG GTGAGCAAGGGCGAGG	IDT	N/A
Primer: *mApple* CE R1: TACAAGGG TGGCGGAGGGTCCGGTGGCGGA GGGTCCGGCGGTGGGGGTTCCGCT AGCATGGATAACGA	IDT	N/A
Primer: *vangl2* CE F1: TCCGCTAG CATGGATAACGAGTCGCAGTACTCG	IDT	N/A
Primer: *mEGFP* CE F1: TCGAATTCA AGGCCTCTCGAGCCACCATGGTG AGCAAGGGCGAG	IDT	N/A
Primer: *mEGFP* CE R1: TCGAATT CAAGGCCTCTCGAGCCACCATGG TGAGCAAGGGCGAG	IDT	N/A
Primer: *prickle2b* CE F1: ATGCCTCTGGAGATGGAGAAGA	IDT	N/A
Primer: *prickle2b* CE R1: CTATAGT TCTAGAGGCTCGAGTCAAGATATAA TACAGTTCTTGCTCTT	IDT	N/A
Primer: *vangl2* CE R1: ACGACT CACTATAGTTCTAGATCACACCGAG GTTTCCGACT	IDT	N/A
Primer: *Dendra2* CE F1: GGATCC CATCGATTCGAATTCGCCACCATGA ACACCCCG	IDT	N/A
Primer: *Dendra2* CE R1: ATGGTG GATCTGAGTCCGGACCACACCTG GCTGGGCAG	IDT	N/A
Primer: *prickle* CE F1: TCCGGACTCAGATCCACCATG	IDT	N/A
Primer: *prickle* CE R1: GGCTCGAG AGGCCTTGAATTCTCACGAGATGAT GCAGTTCTTGTC	IDT	N/A
Primer: *HA-ptprz1b D1902A* F: ACTCAATGGCCTGCCATGGGCGTCCCA	Merck	N/A
Primer: *HA-ptprz1b D1902A* R: TGGGACGCCCATGGCAGGCCATTGAGT	Merck	N/A
Translation-blocking morpholino - *Xenopus* ptprz1 (AUG): 5′-CTTCTAGCAGCGTTTCTTCATG-3′	Gene Tools (USA)	N/A
Standard control morpholino: 5'-CCT CTT ACC TCA GTT ACA ATT TAT A 3'	Gene Tools (USA)	N/A

**Recombinant DNA**		

*pCS2+-mApple-vangl2*	Cloned in this study	*N/A*
*pCS2+-Dendra2-prickle*	Cloned in this study	N/A
*pCS105-EGFP-prickle*	A gift from Brian Ciruna’s lab	N/A
*pCS2+-mEGFP-prickle2b*	Cloned in this study	N/A
*pCS2+-PMT-mApple*	Le et al.^36^	N/A
*HA-ptprz1b*	Le et al.^36^	N/A
HA-ptprz1b D1902A	Cloned in this study	N/A
myc-hVangl1	Belotti et al.^69^	N/A
Flag-hPRICKLE1	Radaszkiewicz et al.^70^	N/A
Flag-hPRICKLE2	Radaszkiewicz et al.^70^	N/A
GFP-xPrickle2 aa 1-893	Butler & Wallingford^68^	N/A
GFP-xPrickle2 1-374 (Pk2 N1)	Cloned in this study	N/A
GFP-xPrickle2 315-605 (Pk2 N2)	Cloned in this study	N/A
GFP-xPrickle2 1-604 (Pk2 N)	Novotna et al.^37^	N/A
GFP-xPrickle2 607-893 (Pk2 C)	Novotna et al.^37^	N/A
GFP-xPrickle2 1-79,400-893 (Pk2 ΔLIM)	Cloned in this study	N/A
Flag-xPrickle2 79-893 (Pk2 ΔPET)	Cloned in this study	N/A
Flag-xPrickle2 314-893 (Pk2 N2+C)	Cloned in this study	N/A
Flag-xPrickle2 400-574 (Pk2 S)	Cloned in this study	N/A

**Software and algorithms**		

ImageJ	Schneider et al.^71^	Version 1.54p
LAX	Leica	Version 3.7.4
GraphPad Prism9	GraphPad	Version 9.0.0
ZEN Blue	Zeiss	Version 3.12
ZEN Black	Zeiss	Version 3.0 SR

**Other**		

LSM900 with Airyscan 2	Zeiss	N/A
Qubit 4 Fluorometer	ThermoFisher	N/A
AxioZoom.V16-Apotome	Zeiss	N/A
Leica SP8 microscope	Leica	N/A
CSU-W1	Yokogawa	N/A

### Experimental model and study participant details

#### HEK293 cells

The HEK293 WT and HEK293 VANGL1^−/−^/2^−/−^ cells^38^ were incubated at 37°C with 5% CO2 in Dulbecco’s modified Eagle medium (cat. no. Ref. 41966-029, Gibco), supplemented with 10% (v/v) fetal bovine serum (lot no. BZ67283PRT, cat. no. 10270, Gibco), and 1% (v/v) penicillin-streptomycin (cat. no. SV30010, Hyclone, Biotech). Wild-type HEK293 cells (ATCC CRL-1573) were obtained from ATCC, whereas HEK293 VANGL1^−/−^/2^−/−^ cells were described previously.^38^ Cell lines were routinely tested for mycoplasma contamination and were confirmed negative. Sample sizes for individual experiments are reported in the corresponding figure legends.

For co-IP, cells were seeded on 6 cm plates with 30% confluency and transfected with 4 μg of plasmid DNA per plate for 24 h (transfected with PEI). For IF, cells were seeded with 30% confluency on 24-well plates with gelatin-coated coverslips and transfected with 0.4 μg of plasmid DNA per well for 24 h (transfected with PEI). For SF, cells were seeded on 15 cm plates with 30% confluency and transfected with 39 μg of plasmid DNA per plate for 24 h (transfected with PEI).

The following plasmids were used for the transfection: HA-Ptprz1b,^36^ HA-Ptprz1b D1902A (cloned in this study), myc-hVANGL1,^69^ Flag-hPRICKLE1,^70^ Flag-hPRICKLE2,^70^ GFP-xPrickle2 aa 1–893 (Pk2 WT),^68^ and GFP-xPrickle2 1–374 (Pk2 N1), GFP-xPrickle2 315–605 (Pk2 N2), GFP-xPrickle2 1–604 (Pk2 N), GFP-xPrickle2 607–893 (Pk2 C), and others, all derived from.^37^ Control DNA plasmid pcDNA3.1 was used to optimize the DNA transfection amount (note: h, human; x, Xenopus; z, zebrafish).

#### Zebrafish

All experiments involving zebrafish were conducted in accordance with protocols (BR22-1497) approved by the Institutional Animal Care and Use Committee (IACUC) of the National University of Singapore. Adult zebrafish were maintained in re-circulating systems under a 14h/10h light/dark cycle at 28°C in the fish facility of the Department of Biological Sciences (DBS) at National University of Singapore.

Wild type (WT) and maternal-zygotic (MZ) *ptprz1b* mutant embryos were used in this study. Sex was not determined because all analyses were performed at embryonic stages prior to sexual differentiation; therefore, sex was not considered a biological variable. Sample sizes were based on standard practices in the field and are reported in the corresponding figure legends.

Both WT and MZ *ptprz1b* mutant embryos were obtained by crossing corresponding adult male and female fish, respectively. Embryos were raised in 0.3X Danieau’s solution (17.4 mM NaCl, 0.21 mM KCl, 0.12 mM MgSO_4_, 0.18 mM Ca(NO_3_)_2_, 1.5 mM HEPES, pH = 7.2) in a 28°C incubator. Embryonic stages were defined by hours postfertilization (hpf) at 28°C and morphological features.^72^

#### Xenopus laevis

All experiments involving *Xenopus laevis* were performed in accordance with Czech legislation governing the use of animals in research and were approved by the relevant institutional and governmental authorities (MSMT-30784/2022 and MSMT-21426/2025, Ministry of Education, Youth and Sports of the Czech Republic; 45055/2020-MZE-18134 and 45980/2023-MZE-13143, Ministry of Agriculture of the Czech Republic; MZP/2025/630/2482, Ministry of the Environment of the Czech Republic).

Embryos were generated and maintained according to established protocols. In short, adult males were anesthetized in 20% MS-222 (Sigma-Aldrich, A5040), and testes were surgically excised and transferred to ice-cold 1× Marc’s Modified Ringer’s solution (MMR; 100 mM NaCl, 2 mM KCl, 1 mM MgSO_4_, 2 mM CaCl_2_, 5 mM HEPES, pH 7.4) supplemented with 50 μg/mL gentamicin (Sigma-Aldrich, G3632). Ovulation in sexually mature females was induced by injection of 260 U human chorionic gonadotropin (hCG; Merck, Ovitrelle 250G) into the dorsal lymph sac approximately 16 h prior to egg collection, with animals maintained overnight at 18°C. Eggs were collected by gentle abdominal pressure and fertilized *in vitro* using freshly macerated testis tissue in 0.1× MMR. Embryos were cultured in 0.1× MMR and staged according to standard developmental tables.^73^

Sex was not determined because all analyses were performed at embryonic stages prior to sexual differentiation; therefore, sex was not considered a biological variable in this study. Sample sizes were based on standard practices in the field and are reported in the corresponding figure legends.

### Method details

#### Co-immunoprecipitation (co-IP)

The next day after transfection, the 6 cm plates were washed with 1 mL of PBS. The lysis buffer was prepared with the following concentrations in order to obtain a cell lysate: 50 mM Tris (pH 7.5), 150 mM NaCl, 1 mM EDTA, 0.5% of NP40, with the addition of 1× protease inhibitor (11836145001, Roche), 1 nM DL-dithiothreitol and 1× phosphatase inhibitor cocktail II (524625, Merck). Then, 1 mL of the lysis buffer was added to each plate and left for 15 min on ice at 4°C. The obtained cell lysates were centrifuged at 14 400 rcf at 4°C for 15 min. From the supernatant were separated two types of samples: 60 μL was kept as the total cell lysate (TCL), and 800 μL was used for the co-IP. In case of double co-IP, 800 μL of lysate was divided into two 400 μL samples. To the TCL samples, 20 μL of 4× Laemmli buffer was added and stored at −20°C until the SDS-polyacrylamide gel electrophoresis (SDS-PAGE) was performed. In the case of IP: Flag, 10 μL of Flag M2 beads (Flag M2 Affinity Gel, A2220-1 ML, Millipore/Merck) were added into the cell lysate and incubated for 3 h at 5°C under agitation. In case of IP: GFP, 4 μL of primary antibody GFP B-2 (sc-9996, Santa Cruz) were added and incubated for 1 h on ice. In case of IP: myc, 4 μL of primary antibody c-myc 9E10 (sc-40, SC Exbio) were added and incubated for 1 h on ice. After incubation of the lysate with primary antibody on ice, 15 μL of the G-protein Sepharose beads (Protein G Sepharose 4 fast flow, 17-0618-01, GE Healthcare) were added and incubated at 4°C overnight under agitation. The beads were previously washed twice with lysis buffer (centrifuged at 0.1 rcf at 4°C for 1 min). After incubation, the samples were washed five times with 800 μL of lysis buffer without all inhibitors (centrifuged for 1 min, 0.1 rcf at 4°C). After removing the supernatant, 33 μL of 2× standard Laemmli buffer was added to each sample. Both IP and TCL samples were boiled (5 min at 95°C) for analysis by SDS-PAGE and continued with WB.

#### Subcellular fractionation (SF) of HEK293 tissue culture cells

The next day after transfection, the 15 cm plates were washed with 10 mL of precooled PBS. The fractionation buffer was prepared with the following concentrations: 20 mM HEPES, 10 mM KCl, 2 mM MgCl_2_, 1 mM EDTA, 1 mM EGTA, adjusted to pH 7.4, with the addition of 1× protease inhibitor (11836145001, Roche), 1 nM DL-dithiothreitol and 1× phosphatase inhibitor cocktail II (524625, Merck). Then, 2 mL of the fractionation buffer was added to each plate and left for 15 min on ice at 4°C. After the incubation, cells were scraped from the plates and incubated for 15 min at 5°C under agitation. The TBS/SDS buffer was prepared with the following concentrations: 50 mM Tris-Cl (pH 7.5), 150 mM NaCl, 0.1% SDS. Next, 100 μL were separated as total cell lysate (TCL), and 100 μL of TBS/SDS buffer was added to the TCL samples. The rest of the cells were centrifuged at 720 rcf at 4°C for 5 min. Supernatant containing mitochondrial, membrane, and cytoplasmic fractions was kept for further procedure. The pellet containing the nuclear fraction was washed with 1 mL of fractionation buffer (centrifuged at 720 rcf at 4°C for 10 min). To the nuclear fraction, 500 μL of TBS/SDS buffer was added. Supernatant containing mitochondrial, membrane, and cytoplasmic fractions was centrifuged at 10 000 rcf at 4°C for 5 min. The supernatant containing the membrane and cytoplasmic fractions was kept for further procedure. The pellet containing mitochondrial fraction was washed with 1 mL of fractionation buffer (centrifuged at 10 000 rcf at 4°C for 10 min). To the mitochondrial fraction, 500 μL of TBS/SDS buffer was added. To the supernatant containing the membrane and cytoplasmic fractions, 1.5 mL of fractionation buffer was added, and it was centrifuged at 100 000 rcf at 4°C for 1 h. The supernatant was kept as a cytoplasmic fraction. The pellet containing the membrane fraction was washed with 3.5 mL of fractionation buffer (centrifuged at 100 000 rcf at 4°C for 1 h). To the membrane fraction, 500 μL of TBS/SDS buffer was added. For the SDS-PAGE and WB analysis, 100 μL of each fraction and TCL was taken, and 33.33 μL of 4× standard Laemmli buffer was added to each sample. TCL and nuclear fraction were sonicated. All samples were boiled (5 min at 95°C) and analyzed by SDS-PAGE, and then continued with WB.

#### Western blotting (WB)

WB membranes were developed using the fusion imaging system (Amersham ECL Prime Luminol Enhancer Solution, 29018903, Cytiva) chemiluminescence documentation system. The primary antibodies used were: HA (ab9110, Abcam—1:1000); Flag (7425, Sigma-Aldrich—1:1000); GFP (20R-GR-011, Fitzgerald—1:1000); myc (C 3956, Sigma—1:1000); Flag M2 (F1804, Sigma/Merck—1:1000); Vangl1 (E−3, SC-166844, Santa Cruz—1:500); pVangl1/2 (AP1206, Abclonal—1:1000); β-actin (4970, Cell Signaling—1:1000); E-cadherin (3195, Cell Signaling—1:1000); β-catenin (610153, BD—1:1000); HSP60 (4870P, Cell Signaling—1:1000); Cox IV (4850P, Cell Signaling—1:1000).

The corresponding secondary antibodies were Anti-Mouse IgG Peroxidase antibody (A4416, Sigma Aldrich—1:5000), and Anti-Rabbit IgG Peroxidase antibody (A0545, Sigma Aldrich—1:5000).

#### Immunofluorescence (IF) microscopy of tissue culture cells

The next day after transfection, cells were washed by PBS and fixed by adding 250 μL of a solution containing 4% of paraformaldehyde for 20 min and blocked in PBTA (3% (w/v) BSA, 0.25% Triton) for 1 h and incubated overnight with primary antibodies (GFP B-2 sc-9996, Santa Cruz—1:250; Flag M2 F1804, Sigma/Merck—1:500; HA Rb ab9110, Abcam—1:500) in PBTA at 4°C. The next day, after washing with PBS, the secondary antibodies (Alexa Fluor 488 Anti-Mouse, A32766, Invitrogen—1:500; Alexa Fluor Anti-Rabbit 568, A11011, Invitrogen—1:500; Cy3 AffiniPure Anti-Rabbit, 711-165-152, Jackson ImmunoResearch—1:500) were incubated in PBTA, washed with PBS, and stained with DAPI (1:1000). All coverslips were mounted on microscopic slides. Cells were visualized using confocal microscopy. Fluorescent confocal images were collected using a Leica SP8 microscope equipped with 63×/1.4 oil objective and LAX software (version 3.7.4).

Quantification of Ptprz1b-Prickle2 signal in HEK293 cells was performed using ImageJ software by the function Plot Profile. Pearson correlation coefficient was calculated using Excel’s function Pearson. Subcellular distribution of C-terminal Prickle2 was quantified in GraphPad Prism9 using one-way ANOVA with Tukey’s post hoc test.

#### DNA construct assembly, capped mRNA synthesis and microinjection of zebrafish embryos

To clone the expression construct for N-terminal tagged mEGFP-Prickle2b (mEGFP-Pk2b), *mEGFP* and zebrafish *pk2b*, CDS DNA fragments were amplified by Phanta Max high fidelity DNA polymerase (Vazyme). The *pCS2+-mEGFP-prickle2b* construct was assembled with *XhoI*-digested *pCS2+*, *mEGFP* and zebrafish *prickle2b* CDS DNA fragments by CloneExpress II One-step cloning kit (Vazyme). For FRAP experiment, *pCS2+-Dendra2-prickle* construct was cloned by assembling *EcoRI*-digested *pCS2+* and DNA fragments of *Dendra2* and *Drosophila prickle* amplified by Phanta Max high fidelity DNA polymerase (Vazyme).

A mMESSAGE mMACHINE SP6 Transcription Kit (ThermoFisher) was used for capped mRNA synthesis with *KpnI*-linearized *pCS105-EGFP-prickle*, *NotI*-linearized *pCS2+-mApple-vangl2*, *NotI*-linearized *pCS2+-Dendra2-prickle*, *MfeI*-linearized *pcs2*+*-mEGFP-pk2b*, *NotI*-linearized *pCS2+-HA-ptprz1b-X4*^*D1902A*^ and *NotI*-linearized *pCS2+-PMT-mApple* plasmids as templates, respectively. The final concentrations of synthesized mRNA were measured by Qubit 4 Fluorometer (ThermoFisher) using Qubit RNA HS Assay Kit (ThermoFisher).

To study Prickle and Vangl2 localization in WT and MZ *ptprz1b* embryos, 100 pg capped mRNA of *mEGFP-prickle2b* (*mEGFP-pk2b*) or *EGFP-prickle* (*EGFP-pk*) was pre-mixed with 40 pg capped mRNA of *mApple-vangl2*, achieving an approximate molar ratio of 1.5 between mEGFP-*Pk2b*/*EGFP-pk* to *mApple*-*vangl2*. The mRNA mixture was then injected into a single cell of 8-cell stage WT and MZ *ptprz1b* mutant embryos, respectively.

To perform FRAP experiments, WT or MZ *ptprz1b* embryos were injected with 100 pg capped mRNA of *Dendra2-prickle* (*Dendra2-pk*) at 1-cell stage. To characterize Dendra2-Pk localization in EVL layers, 100 pg of *Dendra2-pk* and 20 pg of *PMT-mApple* mRNA was injected into a single cell of 8-cell stage WT embryos.

To investigate the effect of Ptprz1b (D1902A) mutant construct, a single cell of 8-cell stage WT embryos was injected with 100 pg of *mEGFP-prickle2b* and 40 pg *mApple-vangl2* mRNA with or without 40 pg of *HA-ptprz1b-X4*^*D1902A*^ mRNA, respectively.

Catalytically inactive variant HA-ptprz1b D1902A was cloned from the HA-ptprz1b plasmid by using dedicated primers and QuikChange II Site-Directed Mutagenesis Kit (200523-5, Agilent).

#### Confocal microscopy and quantitative image analysis of zebrafish embryos

*mEGFP-pk2b/EGFP-pk* and *mApple-vangl2* co-injected WT and MZ *ptprz1b* mutant embryos were mounted with 0.5% low-melting agarose in glass bottom imaging dishes, respectively. Embryos were oriented with the developing dorsal region facing the glass slide of an imaging dish. Single plane fluorescent images of mEGFP-Pk2b/EGFP-Pk and mApple-Vangl2 positive cells were captured at comparable Z-depth from enveloping epithelium using LSM900 confocal microscope (Zeiss) with a 63x (NA = 1.4) oil lens. The same procedure was performed for WT embryos co-injected with *mEGFP-prickle2b*, *mApple-vangl2* mRNA with or without *HA-ptprz1b-X4*^*D1902A*^ mRNA.

To quantitate the relative cytoplasm-localized mEGFP-Pk2b/EGFP-Pk level, two paired regions of interest were drawn on mEGFP-Pk2b/EGFP-Pk and mApple-Vangl2 positive cells using ImageJ.^71^ Circle function was used for cytoplasm ROI, and line function for neighboring membrane ROI. The mean intensities of paired ROIs were measured, and relative cytoplasmic Pk level was calculated by dividing the mean intensity of cytoplasmic ROI by that of membranous ROI. To calculate the relative ratio between plasma membrane localized mEGFP-Pk2b/EGFP-Pk and mApple-Vangl2, the mean intensities of mEGFP-Pk2b/EGFP-Pk and mApple-Vangl2 were measured using the same membranous ROIs. The relative membranous Pk2b/Pk level to Vangl2 was calculated by normalizing the mean intensity of membranous mEGFP-Pk2b/EGFP-Pk to that of membranous mApple-Vangl2, respectively.

To compare the relative Prickle level between WT and MZ *ptprz1b* mutants, statistical analyses were performed using Mann-Whitney test. *p* values were calculated with a confidence interval of 95%.

#### Phalloidin staining of zebrafish embryos

Phalloidin staining was performed based on published protocols.^36^^,^^74^ WT and MZ *ptprz1b* mutant embryos were fixed by 4% PFA/PBST at 10 hpf based on morphological characteristics.^72^ After washing by 1X PBST, embryos were permeabilized by 2% Triton X-100/PBST at room temperature for 2 h. Embryos were subjected to staining by Alexa Fluor 488 Phalloidin (A12379, ThermoFisher) at 4°C overnight according to manufacturer’s instruction. Phalloidin-stained embryos were washed by 1X PBST and counterstained with 5 μg/mL DAPI for 30 min. After a 1X PBST wash, embryos were mounted on imaging glass-bottom dishes with 1.5% (w/w) low melting agarose for imaging on spinning disk confocal (CSU-W1, Yokogawa).

#### FRAP experiments and analysis of zebrafish embryos

At 4 hpf, *Dendra2-pk*-injected WT and MZ *ptprz1b* mutant embryos were mounted with 0.5% low-melting agarose in glass bottom imaging dishes, respectively. FRAP experiments were performed on the apical cortex of EVL cells located at the animal pole, respectively. Five timepoints of pre-bleaching acquisitions, photobleaching and 20 min of timelapse acquisitions during recovery were conducted at comparable Z-planes for both WT and MZ *ptprz1b* embryos. Dendra2-Pk within ROI was photobleached with 488 nm laser until the fluorescent intensity of Dendra2-Pk was below 20% of the pre-bleaching intensity. For pre-bleaching and recovery acquisition, DIC and fluorescent images of Dendra2-Pk positive cells were captured as timelapse series with an interval of 10 s using LSM900 confocal microscope (Zeiss) with a 63x (NA = 1.4) oil lens.

To plot FRAP curves, fluorescent intensity of Dendra2-Pk at each timepoint was background-subtracted and normalized to the intensity of the first pre-bleaching timepoint, respectively.

The mobile fraction (*F*_mobile_) is defined as

$$F_{mobile}=\frac{I_{\text{final}}-I_{\text{post}}}{I_{\text{pre}}-I_{\text{post}}}$$

where *I*_final_ is the average of the final 5 timepoints after photobleaching, I_pre_ is the average fluorescent intensity of the initial 5 pre-bleaching timepoints, and *I*_*post*_ is the average intensity of the first 5 timepoints after photobleaching.

Immobile fraction was calculated as

$$1–F_{mobile}$$

To compare the mobile and immobile fractions between WT and MZ *ptprz1b* mutants, statistical analyses were performed using Mann-Whitney test. *p* values were calculated with a confidence interval of 95%.

#### *Xenopus* loss-of function experiments

For loss-of-function experiments (Figures 5D–5F), *Xenopus* embryos were microinjected into one dorsal blastomere at the 4-cell stage, to achieve unilateral knockdown with the contralateral side serving as an internal control. Embryos were injected in 3% Ficoll 400 (Cytiva, #17-0300-10) prepared in 0.5× MMR using a calibrated microinjection system. Approximately 10 nL of solution containing 2 ng morpholino and a TMR (tetramethylrhodamine) tracer dye was delivered per injection. A translation-blocking morpholino targeting *Xenopus* ptprz1 (AUG) (5′-CTTCTAGCAGCGTTTCTTCATG-3′) or control MO (standard control morpholino) were synthesized by Gene Tools (USA).

Following injection, embryos were allowed to develop to Nieuwkoop and Faber (NF) stage 18 for analysis of anterior neural tube closure and were subsequently fixed in 4% formaldehyde in PBS (Merck). The area of the anterior superficial neural ectoderm was quantified from three independent biological replicates, and results are presented as the ratio between the injected and uninjected sides. The transversal section of the embryo was made using a blade. Embryos were imaged using a Zeiss Axio Zoom V16 equipped with ZEN software. Measurements were performed in ImageJ.

#### Imaging and analysis of *Drosophila* wing hair polarity

Expression of RNAi constructs in the anterior wing compartment was done using the *patched* enhancer-driven Gal4 flies^75^ crossed to TRiP RNAi flies.^76^ Stocks were obtained from the Bloomington Drosophila Stock Center (NIH P40OD018537), UAS-Lar-RNAi (BDSC 43979), UAS-PTP99A-RNAi (BDSC 57299), UAS-Vang-RNAi (BDSC 34354), and ptc-Gal4 (BDSC 2017). Adult wings were dissected and mounted in Hoyer’s solution using standard procedures.^77^ Images were acquired using an upright microscope under visible light.

### Quantification and statistical analysis

Quantitative analyses were performed using ImageJ (NIH), GraphPad Prism 9, and Estimation Stats (www.estimationstats.com). Statistical analyses were performed as indicated in the corresponding figure legends.

For the analysis of Prickle2 subcellular localization in HEK293 cells (Figure 2E′), statistical significance was determined using one-way ANOVA followed by Tukey’s multiple comparisons test. Quantification of Ptprz1b–Prickle2 binding levels (Figure 8A′) and Xenopus neural tube closure experiments (Figure 5E) were analyzed using unpaired two-tailed Student’s t-tests.

For zebrafish experiments (Figures 4D, 4E, 6E, 6F, 7E, 8B′, and 8B″), statistical significance was determined using Mann–Whitney tests with a confidence interval of 95% (CI = 95%). Analysis of notochord width and neuroepithelium depth (Figure 5B) was performed using Estimation Stats with a confidence interval of 95%.

Violin plots display the minimum, 25th percentile, median, 75th percentile, and maximum values of the dataset, with individual observations shown as dots. Data presented in bar graphs are shown as mean ± SD unless otherwise stated. Exact sample sizes, statistical tests, and *p*-values are provided in the corresponding figure legends.
