# Supplementary material for: Ptprz1b phosphatase binds Prickle2 to promote its membrane localization
Source: iScience. 2026 Jul 13;29(8):116707. doi: 10.1016/j.isci.2026.116707 (PMC13382465; doi:10.1016/j.isci.2026.116707)
Supplement: Document S1. Figures S1–S5 [file mmc1.pdf]

iScience, Volume *29*

Supplemental information

Ptprz1b phosphatase binds Prickle2 to promote its membrane localization

Yao Le, Sarka Novotna, Lorena Agostini Maia, Nicholas S. Tolwinski, Christoph Winkler, and Jakub Harnos

Supplementary Figure 1

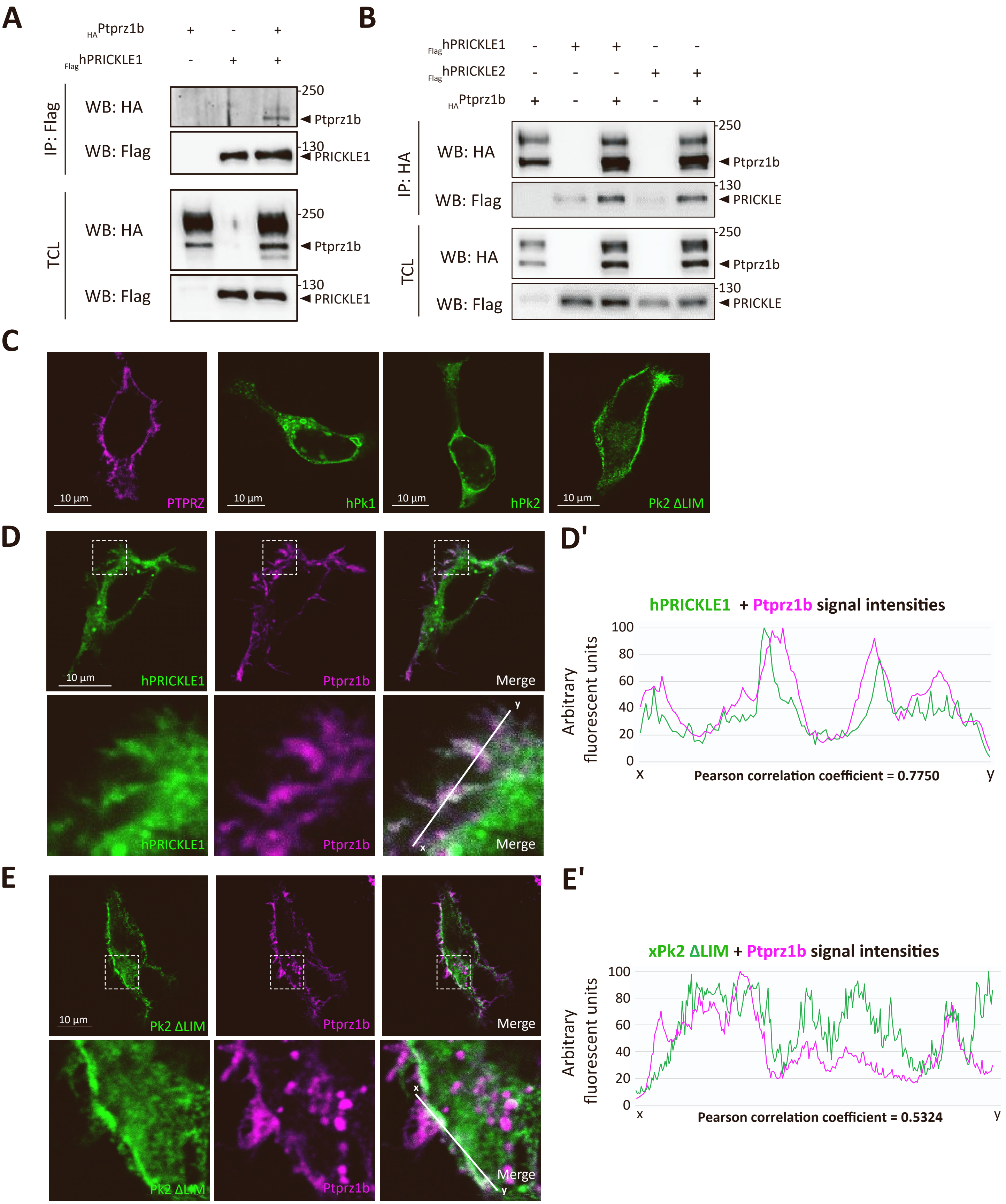

Figure S1. Ptprz1b interacts and co-localizes with Prickle proteins in HEK293 cells, related to Figure 1.
(A) Co-immunoprecipitation of FLAG-tagged human PRICKLE1 with HA-tagged Ptprz1b in HEK293 cells. Input (TCL) and immunoprecipitated (IP: Flag) samples were analyzed by immunoblotting (WB), confirming interaction between Ptprz1b and PRICKLE1. **(B)** Co-immunoprecipitation of HA-tagged Ptprz1b with FLAG-tagged human PRICKLE1 or PRICKLE2 in HEK293 cells. Immunoblot analysis of IP (HA) and TCL samples shows that Ptprz1b interacts with both PRICKLE isoforms. **(C)** Representative confocal images of HEK293 cells expressing Ptprz1b (magenta), hPRICKLE1, hPRICKLE2, or a truncated PK2 ΔLIM construct (green). Scale bars, 10 µm. **(D)** Co-localization of hPRICKLE1 (green) with Ptprz1b (magenta) in HEK293 cells. Insets show magnified regions. Line-scan analysis (white line) is quantified in (D'). **(D')** Fluorescence intensity profiles along the indicated line (x–y) showing co-distribution of hPRICKLE1 and Ptprz1b (Pearson correlation coefficient = 0.7750). **(E)** Co-localization of PK2 ΔLIM (green) with Ptprz1b (magenta) in HEK293 cells. Insets show magnified regions. Line-scan analysis is quantified in (E'). **(E')** Fluorescence intensity profiles along the indicated line (x–y) showing reduced co-localization of PK2 ΔLIM with Ptprz1b (Pearson correlation coefficient = 0.5324).

Supplementary Figure 2

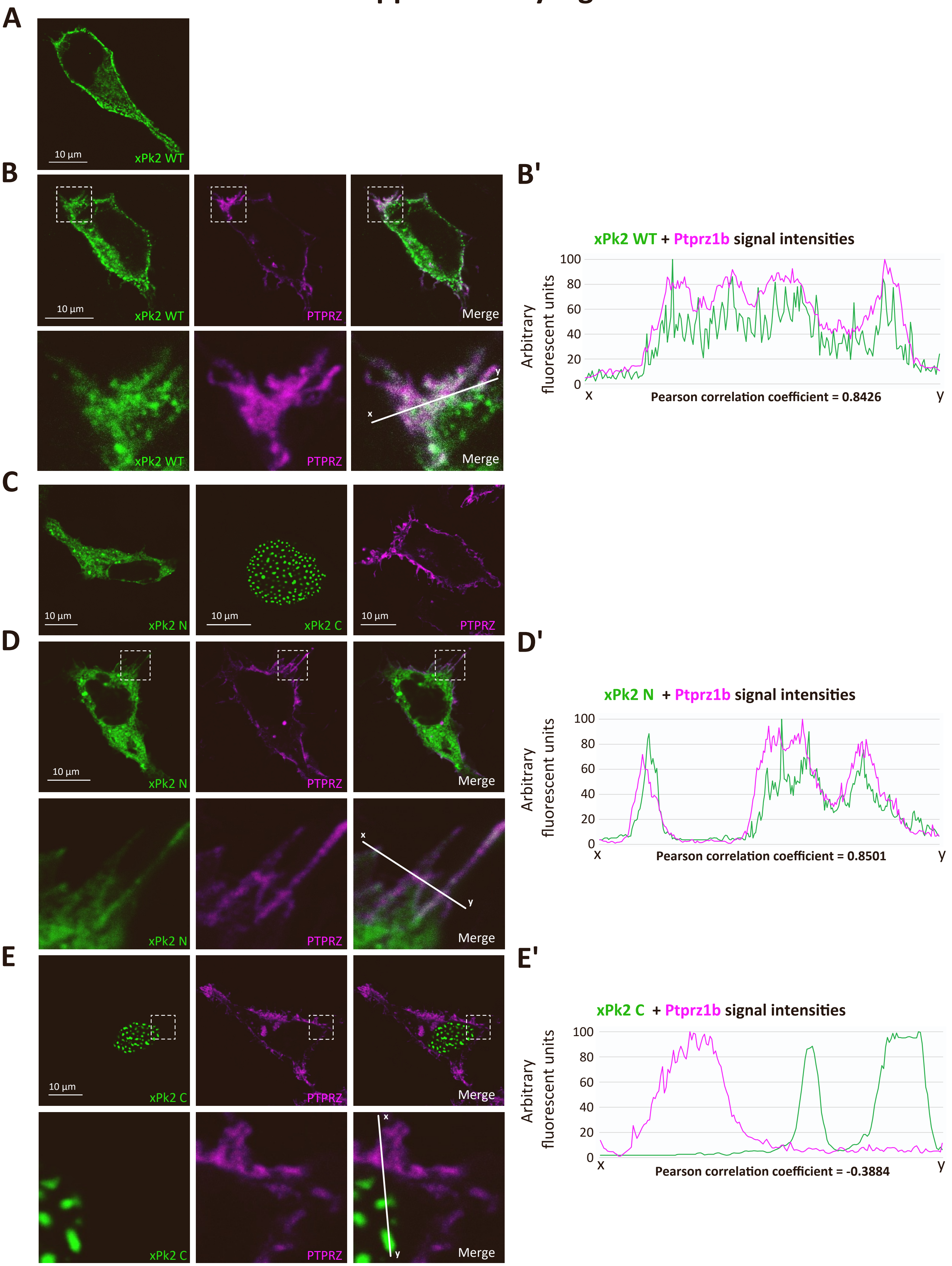

Figure S2. Multiple Prickle2 domains contribute to Ptprz1b co-localization in HEK293 cells, related to Figure 2.
(A) Representative confocal image of xPk2 WT. Scale bar, 10 µm. **(B)** Co-localization of xPk2 WT (green) with Ptprz1b (magenta); inset shows magnified region. Line-scan in (B′). **(B′)** Intensity profiles showing strong co-distribution (Pearson r = 0.843). **(C)** Representative images of xPk2 N-terminal and C-terminal constructs and Ptprz1b. Scale bars, 10 µm. **(D)** Co-localization of xPk2 N with Ptprz1b; inset shows magnified region. Line-scan in (D′). **(D′)** Intensity profiles showing strong co-distribution (Pearson r = 0.850). **(E)** Co-localization of xPk2 C with Ptprz1b; inset shows magnified region. Line-scan in (E′). **(E′)** Intensity profiles showing reduced or no co-localization (Pearson r = −0.388).

Supplementary Figure 3

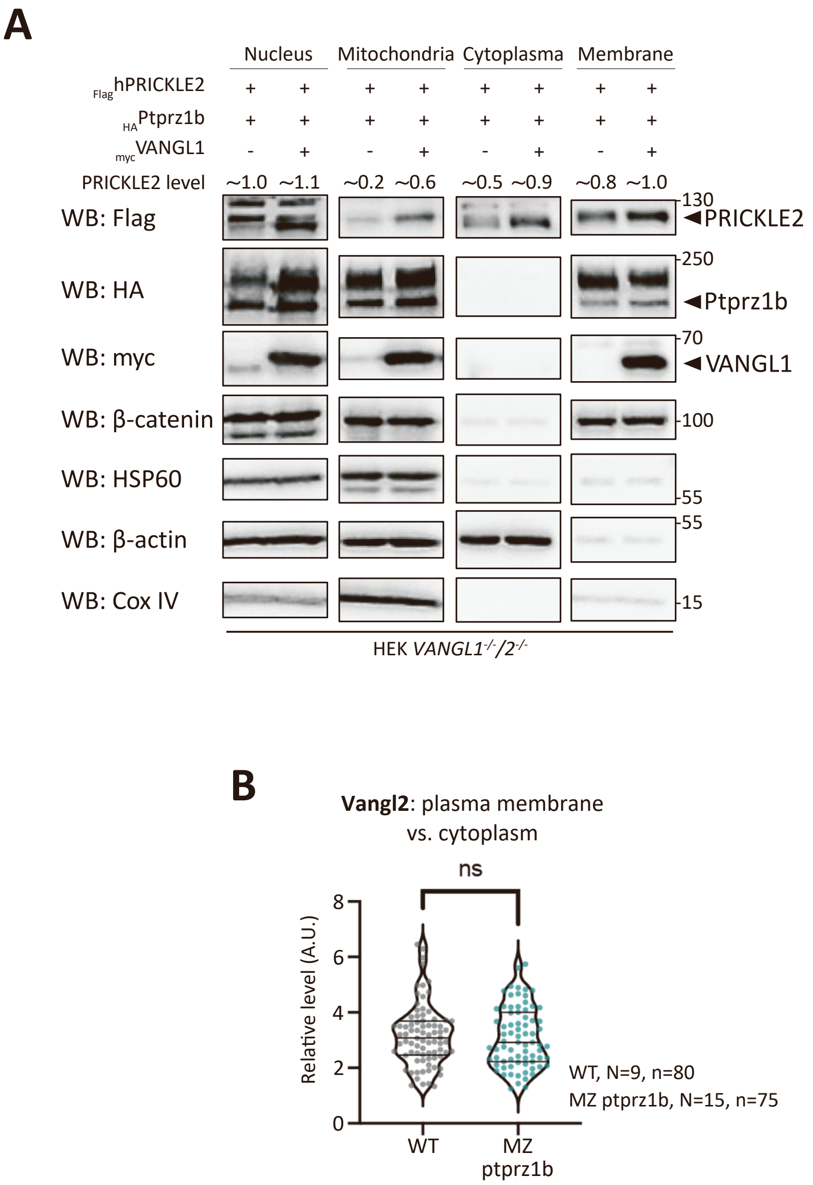

Figure S3. Ptprz1b regulates Prickle2 but not Vangl2 membrane localization, related to Figures 3 and 4.
(A) Subcellular fractionation of HEK293 VANGL1/2⁻/⁻ cells co-expressing hPRICKLE2, Ptprz1b, and Vangl1. Nuclear, mitochondrial, cytoplasmic, and membrane fractions were analyzed by immunoblotting using the indicated markers. PRICKLE2 levels across fractions are shown above. **(B)** Quantification of Vangl2 distribution between plasma membrane and cytoplasm in WT and MZ Ptprz1b cells; no significant difference (ns). Violin plots display the minimum, 25th percentile, median, 75th percentile, and maximum values of the dataset. Individual observations are shown as dots. Related to Figure 4.

Supplementary Figure 4

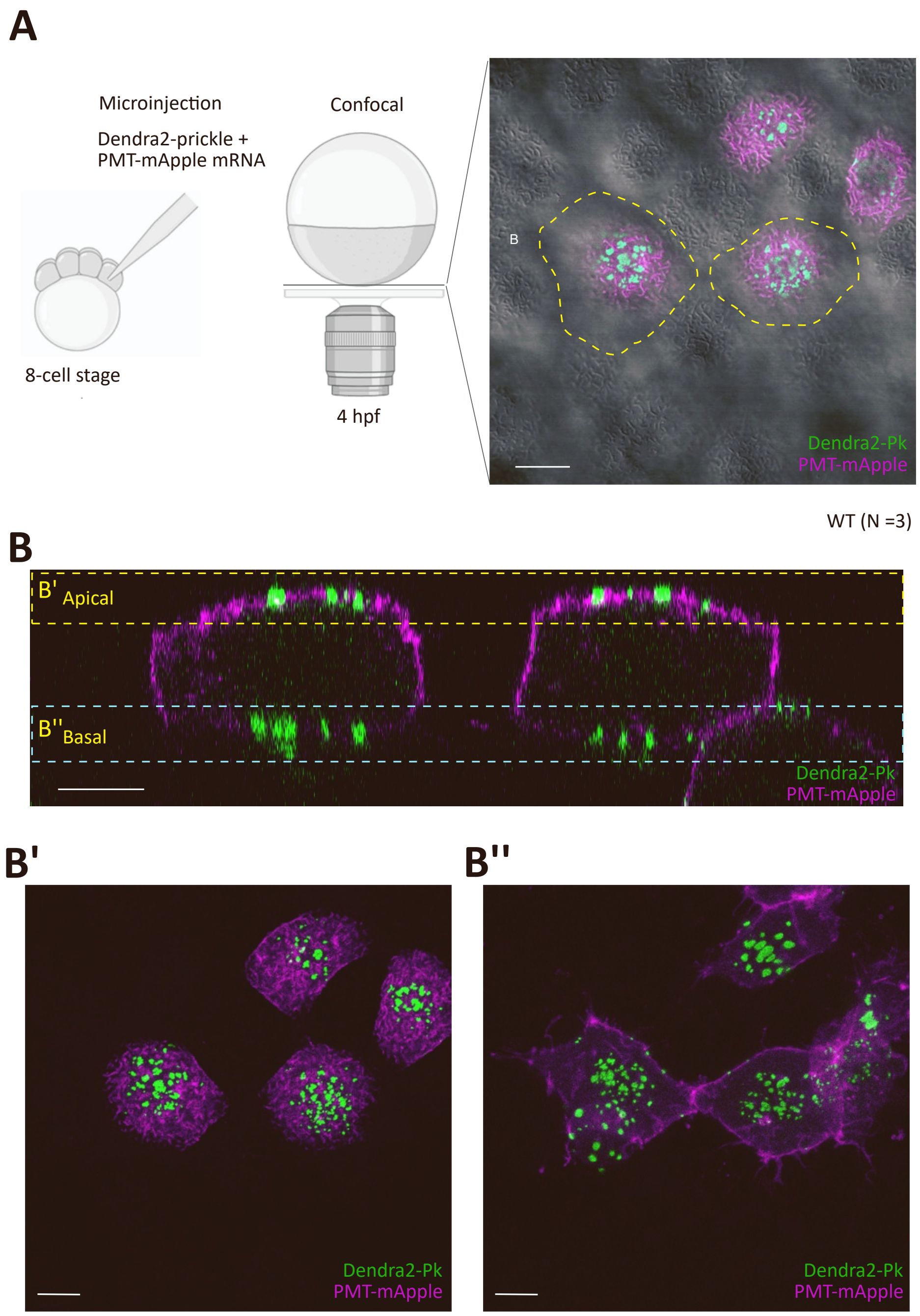

Figure S4. Experimental setup and localization of Dendra2-Prickle during zebrafish FRAP experiments, related to Figure 7.
(A) Demonstration of experimental setup (left two panels). 100 pg of *Dendra2-pk* and 20 pg of *PMT-mApple* mRNA were co-injected into a single cell of 8-cell stage WT embryos. At 4 hpf, embryos were mounted with animal pole facing down and imaged using confocal microscopy. Gray dashed line indicates the Z-plane of acquisition, and the right panel shows representative DIC and fluorescent image of apical cortex of EVL cells acquired at the plane in WT embryos. Yellow dashed lines outline the EVL cells, and white dashed line indicates the position of reconstructed orthogonal view shown in (**B**). Dendra2-EGFP is pseudo-coloured in green and membrane-bound PMT-mApple in magenta. Scale bar = 10 µm. **(B)** Reconstructed orthogonal view of EVL cells expressing Dendra2-EGFP (green) and PMT-mApple (magenta). Dendra2-Pk accumulated as puncta at apical and basal plasma membrane. Yellow and blue dashed squares indicate the Z-range used for maximum intensity projection (MIP) shown in (**B’** and **B’’**). Scale bar = 10 µm.

Supplementary Figure 5

A

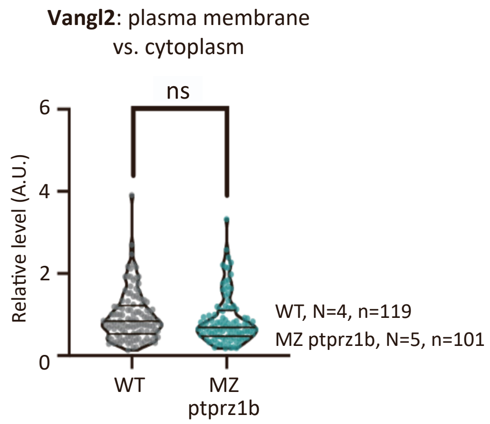

B

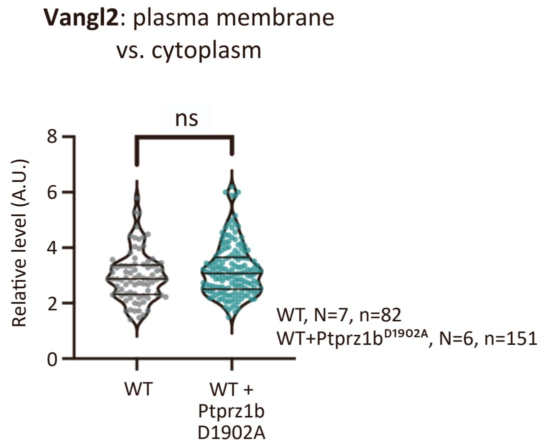

Figure S5. Vangl2 membrane localization is unaffected by loss of Ptprz1b or expression of the Ptprz1b D1902A mutant, related to Figures 6 and 8.

(A) Quantification of Vangl2 distribution between plasma membrane and cytoplasm in WT and MZ Ptprz1b mutant cells; no significant difference (ns). Violin plots display the minimum, 25th percentile, median, 75th percentile, and maximum values of the dataset. Individual observations are shown as dots. Related to Figure 6. **(B)** Quantification of Vangl2 distribution between plasma membrane and cytoplasm in WT and Ptprz1b D1902A; no significant difference (ns). Violin plots display the minimum, 25th percentile, median, 75th percentile, and maximum values of the dataset. Individual observations are shown as dots. Related to Figure 8.
